# Scaling patterns of body plans differ among squirrel ecotypes

**DOI:** 10.7717/peerj.14800

**Published:** 2023-01-25

**Authors:** Tate J. Linden, Abigail E. Burtner, Johannah Rickman, Annika McFeely, Sharlene E. Santana, Chris J. Law

**Affiliations:** 1 University of Washington, Seattle, WA, United States of America; 2 University of Texas at Austin, Austin, TX, United States of America; 3 American Museum of Natural History, New York, NY, United States of America

**Keywords:** Axial skeleton, Body elongation, Ecomorphology, Evolutionary allometry, Thoracolumbar vertebrae

## Abstract

Body size is often hypothesized to facilitate or constrain morphological diversity in the cranial, appendicular, and axial skeletons. However, how overall body shape scales with body size (*i.e.*, body shape allometry) and whether these scaling patterns differ between ecological groups remains poorly investigated. Here, we test whether and how the relationships between body shape, body size, and limb lengths differ among species with different locomotor specializations, and describe the underlying morphological components that contribute to body shape evolution among squirrel (Sciuridae) ecotypes. We quantified the body size and shape of 87 squirrel species from osteological specimens held at museum collections. Using phylogenetic comparative methods, we first found that body shape and its underlying morphological components scale allometrically with body size, but these allometric patterns differ among squirrel ecotypes: chipmunks and gliding squirrels exhibited more elongate bodies with increasing body sizes whereas ground squirrels exhibited more robust bodies with increasing body size. Second, we found that only ground squirrels exhibit a relationship between forelimb length and body shape, where more elongate species exhibit relatively shorter forelimbs. Third, we found that the relative length of the ribs and elongation or shortening of the thoracic region contributes the most to body shape evolution across squirrels. Overall, our work contributes to the growing understanding of mammalian body shape evolution and how it is influenced by body size and locomotor ecology, in this case from robust subterranean to gracile gliding squirrels.

## Introduction

Body size is often hypothesized to be a line of least evolutionary resistance for morphological evolution (Marroig & Cheverud, 2005; Marroig & Cheverud, 2010), and evolutionary changes in body size have a strong influence on an organism’s ecological, physiological, morphological, and functional traits (Schmidt-Nielsen, 1975; LaBarbera, 1989; Calder, 2001; Pyron & Burbink, 2009). Because traits often scale with size, species can adapt to different environments through evolutionary increases or decreases in body size (Zelditch et al., 2017; Zelditch & Swiderski, 2022). However, extrinsic and intrinsic factors often constrain bodies towards certain sizes; therefore, in instances when evolutionary change in body size is limited, new adaptations can arise through evolutionary changes in the shape or proportions of traits (Zelditch et al., 2017). Unsurprisingly, a plethora of work has found that ecological factors affect the evolution of the shape and proportions of the skull (Janis, 1990; Olsen, 2017; Law et al., 2018; Arbour, Curtis & Santana, 2019; Grossnickle et al., 2020; Paluh, Stanley & Blackburn, 2020), limbs (Van Valkenburgh, 1985; Higham et al., 2015; Citadini et al., 2018; Baeckens, Goeyers & Van Damme, 2020) and vertebrae (Buchholtz, 1998; Randau et al., 2016; Jones et al., 2018; Gillet, Frédérich & Parmentier, 2019; Luger et al., 2019; Adler et al., 2022). The evolution of diverse overall body shapes can also facilitate morphological, functional, and ecological innovations that can lead to increased diversification and niche specialization (Wiens, Brandley & Reeder, 2006; Collar et al., 2016; Law, 2019; Friedman, Price & Wainwright, 2021; Morinaga & Bergmann, 2020).

Although the morphological patterns of body shape evolution are well-studied in vertebrates, including squamate reptiles (Wiens & Slingluff, 2001; Bergmann et al., 2020; Grinham & Norman, 2020), fishes (Strauss, 1985; Mehta et al., 2010; Ward & Mehta, 2010; Friedman et al., 2019), and, more recently, carnivoran mammals (Law, 2021a; Law, 2022), few have investigated evolutionary allometry between body shape and size. In Indo-Pacific shore fishes, body size explains 3–50% of body shape variation depending on taxonomic families, and larger fishes tend to exhibit more elongate bodies (Friedman et al., 2019). Similarly, in carnivoran mammals, allometric effects of body size influence body shape variation (Law, 2021a). However, the boundary between terrestrial and aquatic habitats affects these allometric patterns: like fishes (Friedman et al., 2019), aquatic carnivorans tend to evolve more elongate bodies with increasing size (Law, 2021b) whereas terrestrial carnivorans tend to evolve more robust bodies with increasing size. This suggests that body shape allometries differ between locomotor ecologies. Elongate body shapes are associated with fin and limb size reduction (Gans, 1975; Wake, 1991; Wiens & Slingluff, 2001; Skinner, Lee & Hutchinson, 2008; Ward & Mehta, 2010). In tetrapods, researchers have found that the forelimbs are generally reduced or lost prior to the hind limbs through evolutionary time (Gans, 1975; Wiens & Slingluff, 2001; Brandley, Huelsenbeck & Wiens, 2008; Law, Slater & Mehta, 2019; Morinaga & Bergmann, 2020; but see Kohlsdorf & Wagner, 2006; Bergmann & Morinaga, 2019). How locomotor ecologies affect relationships between body shape and limb lengths in mammals remains to be tested.

Despite observed convergence in body plans (*e.g.*, Brandley, Huelsenbeck & Wiens, 2008; Friedman et al., 2016; Bergmann & Morinaga, 2018; Morinaga & Bergmann, 2020; Law, 2022), similar body shapes can evolve through multiple pathways including elongation of the head, reduction of body depth, and elongation of the body axis *via* changes in total vertebral number and/or elongation of individual vertebrae (Parra-Olea & Wake, 2001; Ward & Brainerd, 2007; Ward & Mehta, 2010; Collar et al., 2016; Law, 2022). Because vertebral number is constrained in most mammals (Narita & Kurutani, 2005), they evolve more elongate or robust bodies through changes in body depth and/or elongation or shortening of the skull and vertebrae rather than vertebral number. For example, the elongated neck found in giraffes is due to elongation of the cervical vertebrae rather than increase in the number of vertebrae (Badlangana, Adams & Manger, 2009; Danowitz, Domalski & Solounias, 2015; Danowitz et al., 2015). In carnivorans, the elongation or shortening of the thoracolumbar regions and changes in rib lengths contribute most to variation in body shape, ranging from elongate weasels to robust bears (Law, 2021b). Whether these patterns are similarly found in other mammalian clades is not known.

In this study, we used squirrels (family Sciuridae) as a model system to examine the effects of body size on body shape evolution. We also investigated the relationship between body shape and limb length as well as the underlying morphological components that contribute to body shape evolution. Squirrels are qualitatively diverse, with body sizes ranging from 32 g least chipmunks to 8 kg Olympic marmots and body shapes ranging from the rotund bodies of marmots to the lithe bodies of gliding squirrels. In addition to their diverse body plans, squirrels exhibit varied locomotor ecologies and habitat use, including four ecotypes: ground squirrels that dig, tree squirrels that climb, gliding squirrels that glide between trees, and more versatile chipmunks that can dig and climb. Therefore, we also examined how morphological patterns differ between these four squirrel ecotypes.

Our objectives were three-fold. First, we examined the relationships between body size and body shape and between body shape and limb lengths. Second, we tested if these relationships differed between ecotypes. We predicted that ground squirrels would exhibit more robust bodies with increasing body size and relatively shorter limbs. These phenotypes would provide more structural support and force production when digging large tunnel systems (Samuels & Van Valkenburgh, 2008). We predicted that all other ecotypes (*i.e.,* chipmunks, tree squirrels, and gliding squirrels) would evolve more elongate bodies with increasing body size. Elongate bodies could facilitate heightened flexibility and maneuverability for large squirrels when navigating complex microhabitats such as tree branches. While it is known that the forelimbs of gliding squirrels are relatively longer than those of other ecotypes (Peterka, 1936; Bryant, 1945; Thorington & Heaney, 1981; Grossnickle et al., 2020), the relationship between body shape and forelimb length remains unstudied. Accordingly, we predicted that gliding squirrels would exhibit relatively longer forelimbs with increasing body elongation to increase patagium surface area for gliding. In contrast, we predicted that chipmunks and tree squirrels would exhibit relatively shorter forelimbs with increasing body elongation following similar patterns found in terrestrial carnivoran mammals (Law, Slater & Mehta, 2019). For our final objective, we examined which cranial and axial components contributed the most to overall body shape evolution across squirrels and within each ecotype. We predicted that thoracolumbar elongation or shortening will be the biggest contributor to body shape evolution, as this region provides the body’s primary structural support against gravity (Kardong, 2014).

## Material and Methods

### Quantifying squirrel body shape

We quantified squirrel body shape using 220 osteological specimens across 87 species. These specimens were sourced from the collections of 11 museums (Table S1). We used female, male, and sex-unknown individuals for our measurements in order to achieve the largest sample size possible per species and across species. Additionally, each specimen measured was fully mature, which we determined by verifying that the cranial exoccipital-basioccipital and basisphenoid-basioccipital sutures were fused and that all vertebrae and limb bones were ossified.

**Figure 1:**
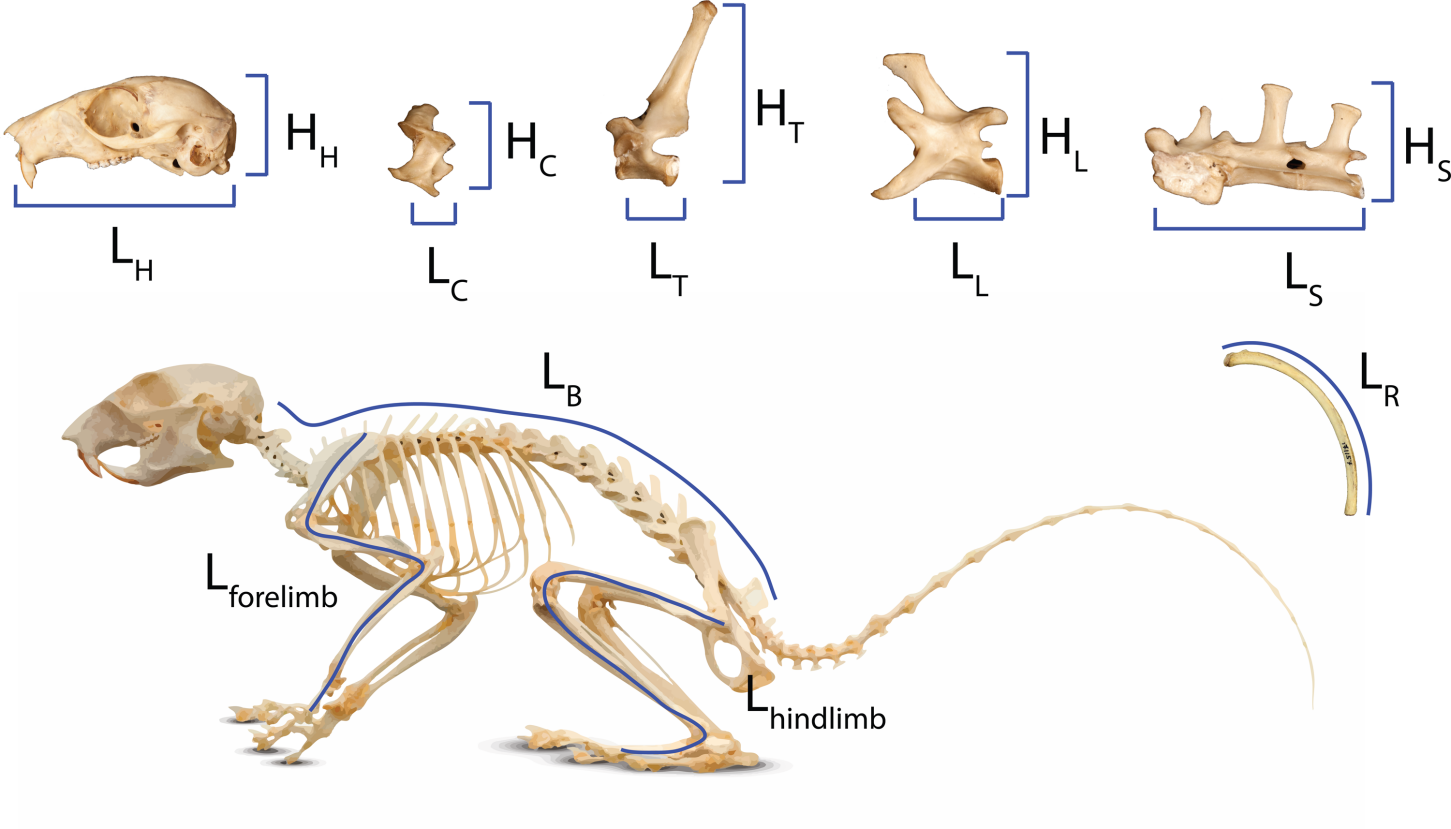
Measurements of body regions used to calculate head-body elongation ratio (hbER), head ER, and axial elongation index (AEI) of the cervical, thoracic, lumbar, and sacral regions. Measurements of body regions used to calculate head-body elongation ratio (hbER), head ER, and axial elongation index (AEI) of the cervical, thoracic, lumbar, and sacral regions. *L*_*X*_ = lengths and *H*_*X*_ = heights. hbER = (*L*_*H*_ + *L*_*B*_)/*L*_*R*_ *AEI*_*V*_ = ∑ *L*_*V*_/mean(*H*_*V*_). H = head; R = rib; C = cervical; T = thoracic; L = lumbar; S = sacral; B = body length.

We used the head-body elongation ratio (hbER) as our metric of body shape (Law, Slater & Mehta, 2019), which was calculated as the sum of head length (L_*H*_) and body length (L_*B*_) divided by the body depth (L_*R*_): hbER = (L_*H*_ + L_*B*_)/L_*R* _ (Fig. 1). We measured head length as the condylobasal length of the cranium from the anteriormost point on the premallixa to the posteriormost point on the surfaces of the occipital condyles. We estimated body length by summing the centrum lengths (measured along the ventral surface of the vertebral centrum) of each cervical, thoracic, lumbar, and sacral vertebrae. All linear measurements were taken to the nearest 0.01 mm using digital calipers. We estimated body depth as the average length of the four longest ribs. Each rib was measured as a curve from the end of the capitulum to the point of articulation with the costal cartilage using a flexible measuring tape.

We also quantified the underlying cranial and axial components that contribute to body shape evolution. Head elongation ratio (head ER) was calculated by dividing cranial length (L_*H*_) by cranial height (H_*H*_). We used a modified version of the axial elongation index (AEI; Ward & Brainerd, 2007; Law, Slater & Mehta, 2019) to examine how each vertebral region (*i.e.,* cervical, thoracic, lumbar, and sacral) contributes to body shape evolution. For each vertebral region (V), we calculated AEI_*V*_ as the total sum of vertebral lengths (L_*V*_ measured along the ventral surface of the vertebral centrum) divided by the average vertebral height (H_*V*_; measured from the ventral surface of the centrum to the tip of the neural spine): AEI_*V*_ = ∑ L_*V*_/mean(H_*V*_). We quantified body size using the geometric mean of our linear measurements (Nth root of the product of our measurements for the cranium, vertebrae, and ribs; N = 11) (Mosimann, 1970; Klingenberg, 2016).

Lastly, we measured the lengths of the forelimb and hind limb. Forelimb length was estimated by summing the lengths of the scapula (from the dorsalmost point on the glenoid fossa to the ventralmost point on the inferior angle), humerus (from the dorsalmost point on the humeral head to the ventralmost point on the capitulum), radius (from the dorsalmost point on the radial head to the ventralmost point on the styloid process), and the third metatarsals (from the distalmost point on the metatarsal head to the proximalmost point on the metatarsal base). Hind limb length was estimated by summing the lengths of the femur (from the dorsalmost point on the femoral neck to the ventralmost point on the patellar surface), tibia (from the dorsalmost point on the intercondylar eminence to the ventralmost point on the articular surface), and third metacarpal (from the distalmost point on the metacarpal head to the proximalmost point on the metacarpal base). Carpals and tarsals were not measured because they are frequently missing. To increase species sample sizes, we used reduced limb datasets in which total limb lengths consisted of just the long bones (*i.e.,* humerus and radius for the forelimb and femur and tibia for the hind limb) and the metatarsals and metacarpals removed. Our findings with these datasets indicated that the major patterns remain largely the same (see Results).

### Ecotype data

**Figure 2:**
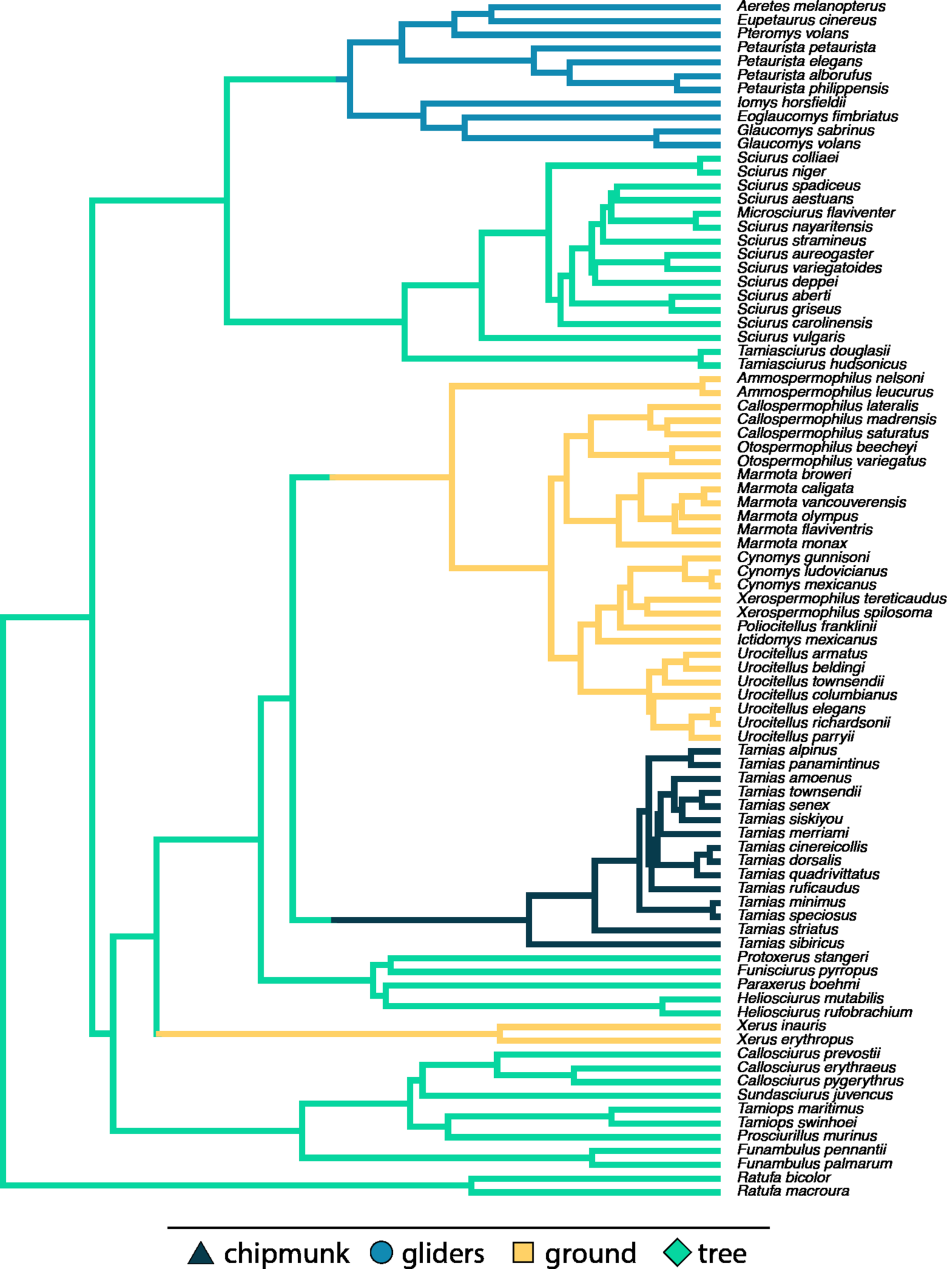
Pruned phylogeny of studied species with branch colors representing ecotypes.

We categorized species into four ecotypes: chipmunks (*n* = 15), gliding squirrels (*n* = 11), ground squirrels (*n* = 29), and tree squirrels (*n* = 32) (Fig. 2), representing all ecotypes found in Sciuridae. We categorized squirrels based on locomotion and nest location using natural history information from the *Handbook of the Mammals of the World* (Wilson, Lacher & Mittermeier, 2016) and the Animal Diversity Web (https://animaldiversity.org/). We classified tree squirrels as species that nest in trees and display both arboreal and scansorial locomotion, gliding squirrels as species with derived morphologies (*i.e.,* patagia) for gliding locomotion, and ground squirrels as species that nest in underground burrows and display fossorial locomotion (Hayssen, 2008). Our fourth ecotype group was chipmunks (genus *Tamias*), which display the broadest range of locomotor and nesting behaviors; species are considered terrestrial, semi-fossorial, or semi-arboreal depending on the source consulted, but none are considered fully fossorial or arboreal.

### Statistical analyses

All analyses were performed under a phylogenetic framework using Upham, Esselstyn & Jetz (2019) recent phylogeny of mammals pruned to include just the 87 studied squirrels. We took the natural logarithm of all traits prior to statistical analyses and performed all analyses in R 4.2.2 (R Core Team, 2022).

We tested for allometric relationships between body shape and body size as the explanatory variable using a phylogenetic generalized least squares (PGLS) regression with the R package phylolm v2.6.2 (Tung Ho si & Ané, 2014). We then tested if body shape allometry differed among ecotypes using a PGLS regression with an ANCOVA design with Type II sum of squares: body shape ∼ body size*ecotype. To determine if there was significant body shape allometry, we generated 95% confidence intervals using bootstrapping (1,000 replications) of the slopes and intercepts of the model. Confidence intervals that deviated from an isometric slope of 0 were interpreted as exhibiting significant positive allometry (slope > 0) or negative allometry (slope < 0). The isometric slope was set as 0 because hbER is a dimensionless ratio. Additionally, we determined whether the allometric slopes differed between ecotypes by comparing the mean slope of each ecotype with the 95% confidence intervals of the mean slope of the other ecotypes. All regression coefficients were simultaneously estimated with phylogenetic signal in the residual error as Pagel’s lambda (λ).

We examined if the relationships between limb length and body shape using PGLS and tested if these relationships differed among ecotypes using PGLS regressions with an ANCOVA design in phylolm. We excluded 11 species from which we were unable to collect limb data. We determined if each ecotype exhibited a significant limb ∼ body shape relationship based on whether the 95% confidence intervals from 1,000 bootstrap replications deviated from an isometric slope of 0. We size-corrected limb lengths prior to analyses by extracting residuals for each trait against the geometric mean using a PGLS. We also tested if size-corrected limb lengths differed among ecotypes. Differences among ecotypes were considered significant in each measure of limb length if an ecotype’s value for mean limb length was outside of other ecotypes’ 95% bootstrap confidence intervals for that measure of limb length. We also ran PGLS regressions on the full limb datasets that included the metacarpals and metatarsals.

Lastly, we determined which morphological components (*i.e.,* cranium, ribs, cervical vertebrae, thoracic vertebrae, lumbar vertebrae, and sacrum) were most related to body shape by performing phylogenetic multiple regressions with the R package RRPP v1.0.0 (Collyer & Adams, 2018). The six morphological components were used as explanatory variables and body shape was used as the response variable. We used R^2^ to examine the proportion of the variance in body shape explained by all the morphological components and determined that the component most associated with body shape had the highest R^2^. Statistical significance was determined using the random residual permutation procedure (RRPP) with 1,000 iterations (Adams & Collyer, 2018). We performed phylogenetic multiple regressions for the whole clade as well as each of the four ecotypes.

## Results

### Body shape allometry

**Figure 3:**
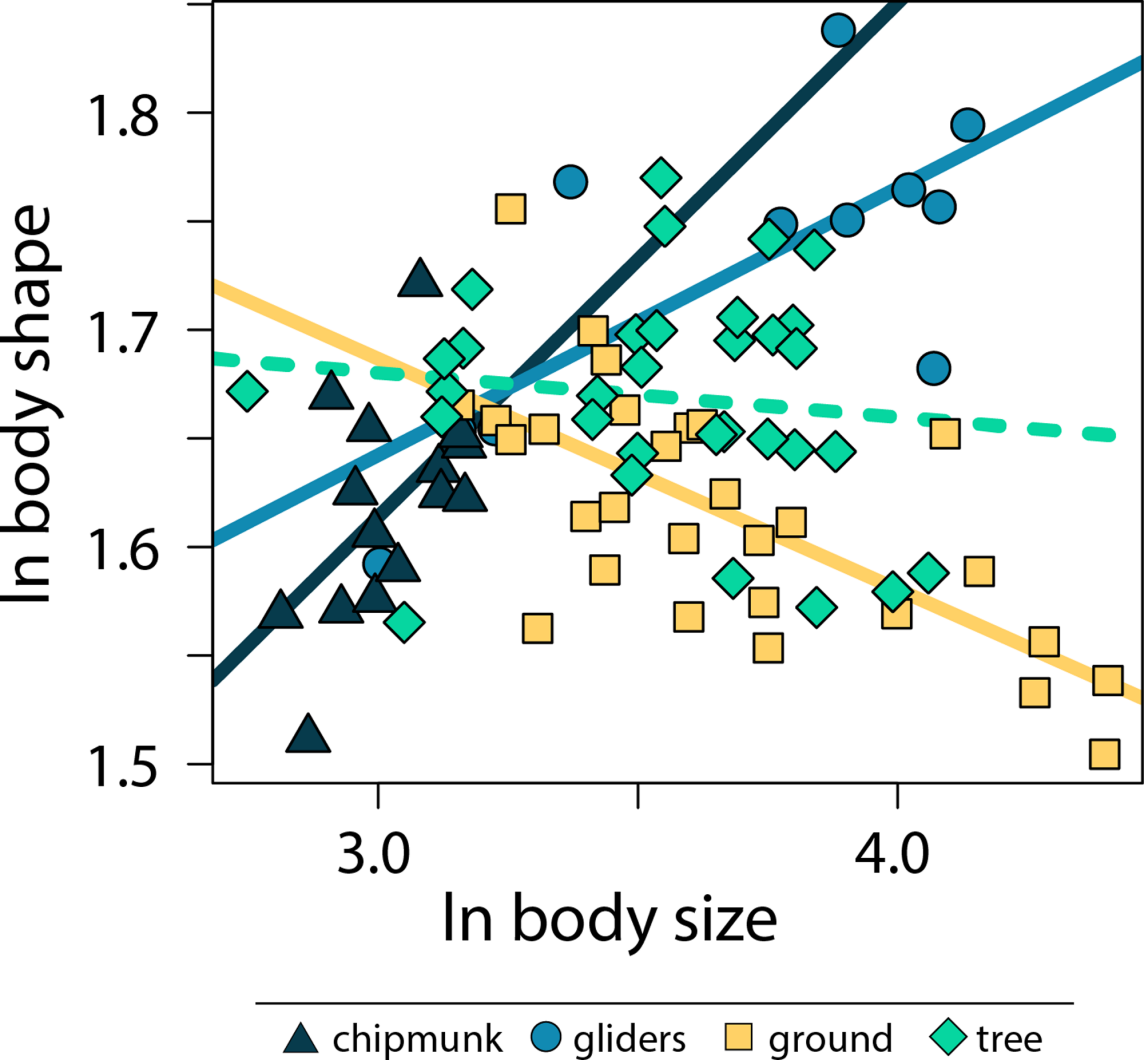
Scatter plot of ln body size and ln body shape. Body size was quantified as the geometric mean of cranial and all axial measurements, and body shape was quantified using the head-body elongation ratio. Relationships between body size and body shape were tested using PGLS with an ANCOVA design. Solid lines indicate significant relationships, and dashed lines indicate non-significant relationships. Confidence intervals that deviated from an isometric slope of 0 were interpreted as exhibiting significant positive allometry (slope > 0) or negative allometry (slope < 0).

Across all squirrels, we found that there was no significant relationship between body size and body shape (adjusted R^2^ < 0.01, λ = 0.80, slope [95% CI] = −0.02 [−0.07:0.02]; Tables S2, S3). However, including body size * ecotype as an interaction term indicated that allometric trends in body shape differed between ecotypes (adjusted R^2^ = 0.50, λ = 0.00; Fig. 3; Tables S2, S3). Gliding squirrels (0.12 [0.06:0.19]) and chipmunks (0.24 [0.03:0.46]) exhibited positive allometry, indicating that gliding and chipmunk species evolved more elongate bodies with increasing body size. In contrast, ground squirrels (−0.11 [−0.15:−0.06]) exhibited negative allometry, indicating that ground squirrels evolved more robust bodies with increasing body size. Tree squirrels did not exhibit a significant relationship between body shape and body size (−0.02 [−0.08:0.03]).

Within the body shape components, head ER (adjusted R^2^ = 0.34, λ = 0.67; 0.18 [0.13:0.23]) and cervical AEI (adjusted R^2^ = 0.54, λ = 0.86; 0.22 [0.18:0.26]) scaled with positive allometry with body size whereas lumbar ER (adjusted R^2^ = 0.05, λ = 0.91; −0.09 [−0.16:−0.02]) scaled with negative allometry across all squirrels (Tables S2, S3). These results indicate that squirrels exhibit more elongate heads and cervical regions but more robust lumbar regions with increasing body size. In contrast, thoracic and sacral regions and size-corrected rib length did not scale with body size (Tables S2, S3).

**Figure 4:**
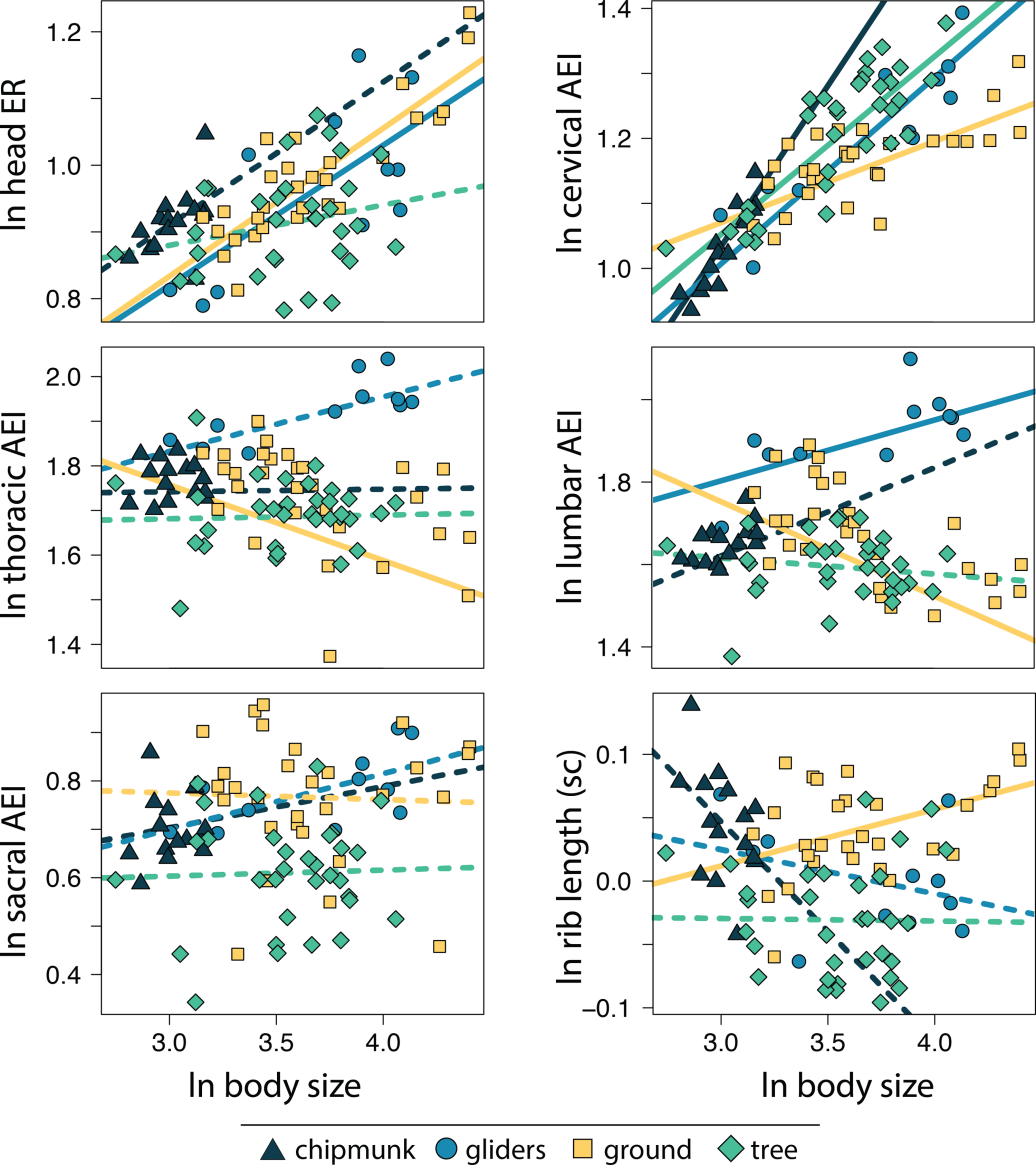
Scatter plot of ln body size and ln each morphological component underlying body shape. Body size was quantified as the geometric mean of cranial and all axial measurements. Relationships between body size and each morphological component were tested using PGLS with an ANCOVA design. Confidence intervals that deviated from an isometric slope of 0 were interpreted as exhibiting significant positive allometry (slope > 0) or negative allometry (slope < 0). Solid lines indicate significant relationships, and dashed lines indicate non-significant relationships.

Allometric trends in body shape components also differed among ecotypes. For gliding squirrels, we found positive allometry for ln head ER (0.21 [0.12:0.30]), ln cervical AEI (0.29 [0.20:0.37]), and ln lumbar AEI (0.15 [0.01:0.29]; Fig. 4; Tables S2, S3). Tree squirrels exhibited a positive slope for only ln cervical AEI (0.27 [0.20:0.35]). Ground squirrels showed negative allometry for ln thoracic AEI (−0.17 [−0.27:−0.09]) and ln lumbar AEI (−0.23 [−0.32:−0.14]) but positive allometry for ln cervical AEI (0.13 [0.07:0.18]), ln head ER (0.22 [0.15:0.29]), and ln size-corrected rib length (0.05 [0.00:0.08]). Chipmunks showed a positive trend for ln cervical AEI (0.50 [0.31:0.67]). We found no significant allometry for ln sacral AEI in any ecotype (Fig. 4; Tables S2, S3).

### Relationships between body shape and limb length

Across all squirrels, we found that body shape did not scale with either size-corrected forelimb length (adjusted R^2^ < 0.01, λ = 0.92, slope [95% CI] = 0.08 [−0.17:0.34]) or size-corrected hind limb length (adjusted R^2^ = 0.20, λ = 0.91, 0.08 [−0.17:0.29]; Tables S4, S5). Findings remained largely the same when using the full forelimb (scapula + humerus + radius + metacarpal) and hind limb (femur + tibia + metatarsal) datasets (Tables S4, S5).

**Figure 5:**
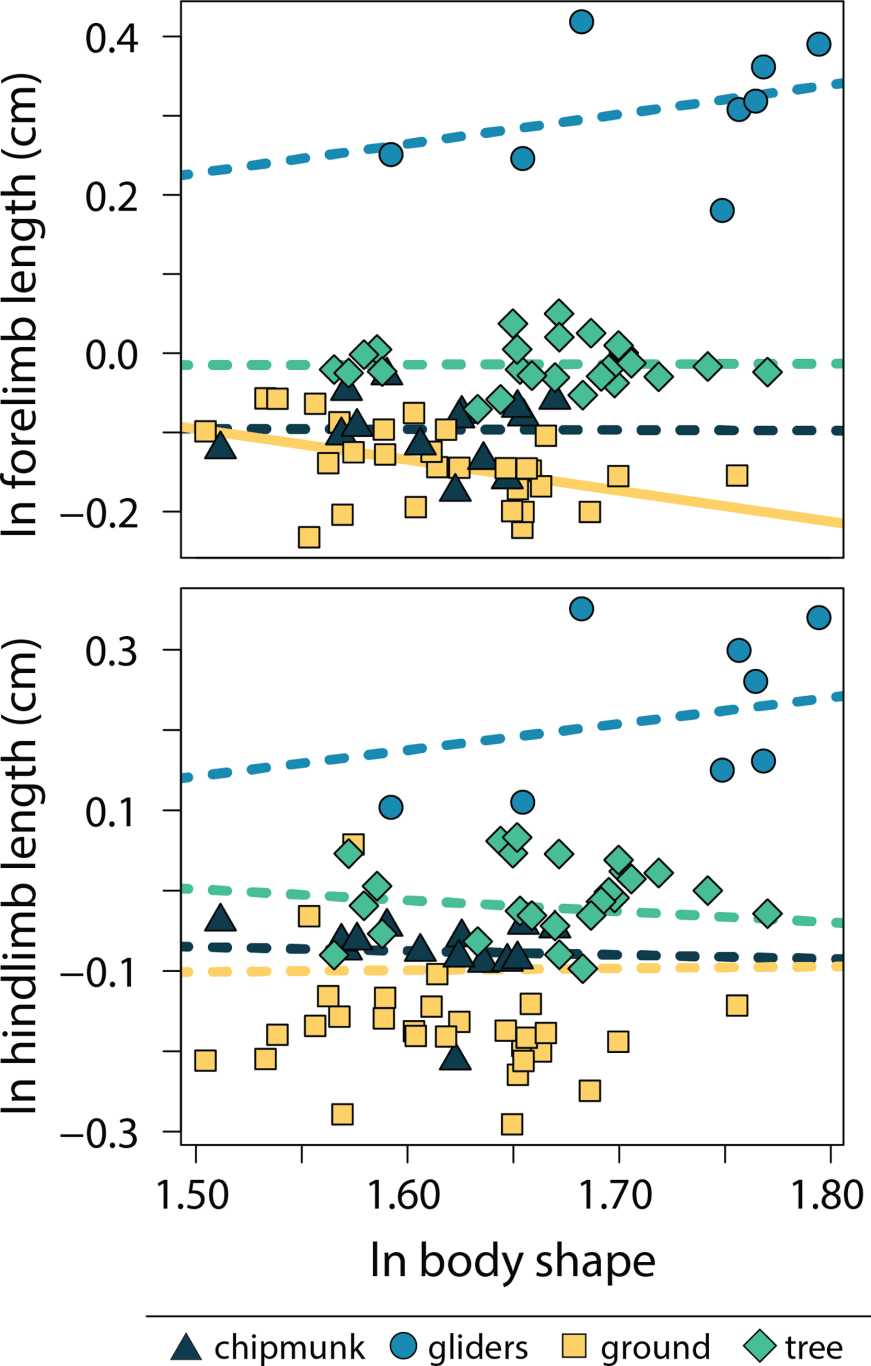
Scatter plot of ln body shape and ln size-corrected forelimb and hind limb lengths. Body shape was quantified using the head-body elongation ratio. Relationships between body shape and limb lengths were tested using PGLS with an ANCOVA design. Confidence intervals that deviated from an isometric slope of 0 were interpreted as exhibiting significant positive relationship (slope > 0) or negative relationship (slope < 0). Solid lines indicate significant relationships, and dashed lines indicate non-significant relationships.

Relationships between body shape and size-corrected forelimb lengths differed between ecotypes (adjusted R^2^ = 0.90, λ = 0.00; Tables S4, S5). Ground squirrels exhibited a negative relationship between size-corrected forelimb length and body shape (−0.391 [−0.672:−0.098]), whereas the remaining ecotypes did not exhibit significant relationships between relative forelimb length and body shape (chipmunk slope = −0.01 [−0.56:0.48]; gliding squirrel slope = 0.37 [−0.04:0.86]; tree squirrel slope = 0.01 [−0.32:0.32]). In contrast, none of the ecotypes exhibited significant relationships between relative hind limb length and body shape (adjusted R^2^ = 0.18, λ = 0.91; chipmunk slope = −0.05 [−0.45:0.32]; gliding squirrel slope = 0.33 [−0.35:09]; ground squirrel slope = 0.02 [−0.31:0.34]; tree squirrel slope = −0.14 [−0.55:0.25]; Fig. 5).

**Figure 6:**
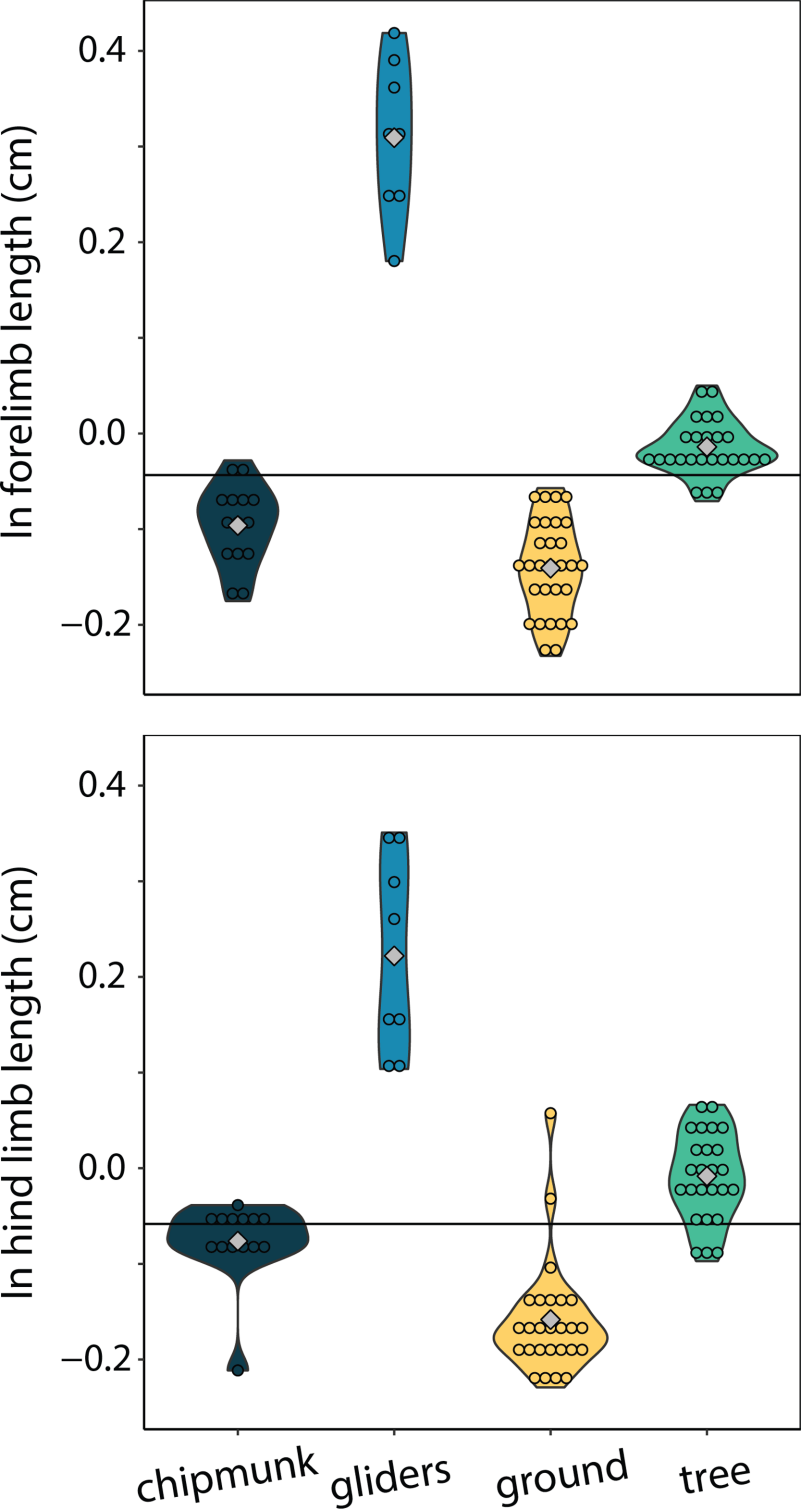
Violin plots of size-corrected forelimb length and size-corrected hind limb length. Gliding squirrels exhibited relatively longer forelimbs than all other ecotypes and relatively longer hind limbs than ground squirrels. The horizontal black line indicates the mean size-corrected limb length for all ecotypes.

Only gliders exhibited significantly different relative forelimb lengths. Gliding squirrels exhibited relatively longer forelimbs (residuals [95% CI] = 0.49 cm [0.17:0.80 cm]) than all other ecotypes (chipmunks = 0.07 cm [−0.23:0.36 cm]; ground squirrels = 0.03 cm [−0.27:0.32 cm]; tree squirrels = 0.16 cm [−1.45:0.46 cm]) (adjusted R^2^ = 0.00, λ = 0.91; Fig. 6). There were no significant differences in relatively longer hind limbs among chipmunks (−0.07 cm [−0.42:0.26 cm]), gliding squirrels (0.23 cm [−0.17:0.58 cm]), ground squirrels (−0.09 cm [−0.43:0.22 cm]), and tree squirrels (−0.01 cm [−0.37:0.32 cm]) (adjusted R^2^ = 0.20, λ = 0.91; Fig. 6).

### Morphological components underlying body shape

**Figure 7:**
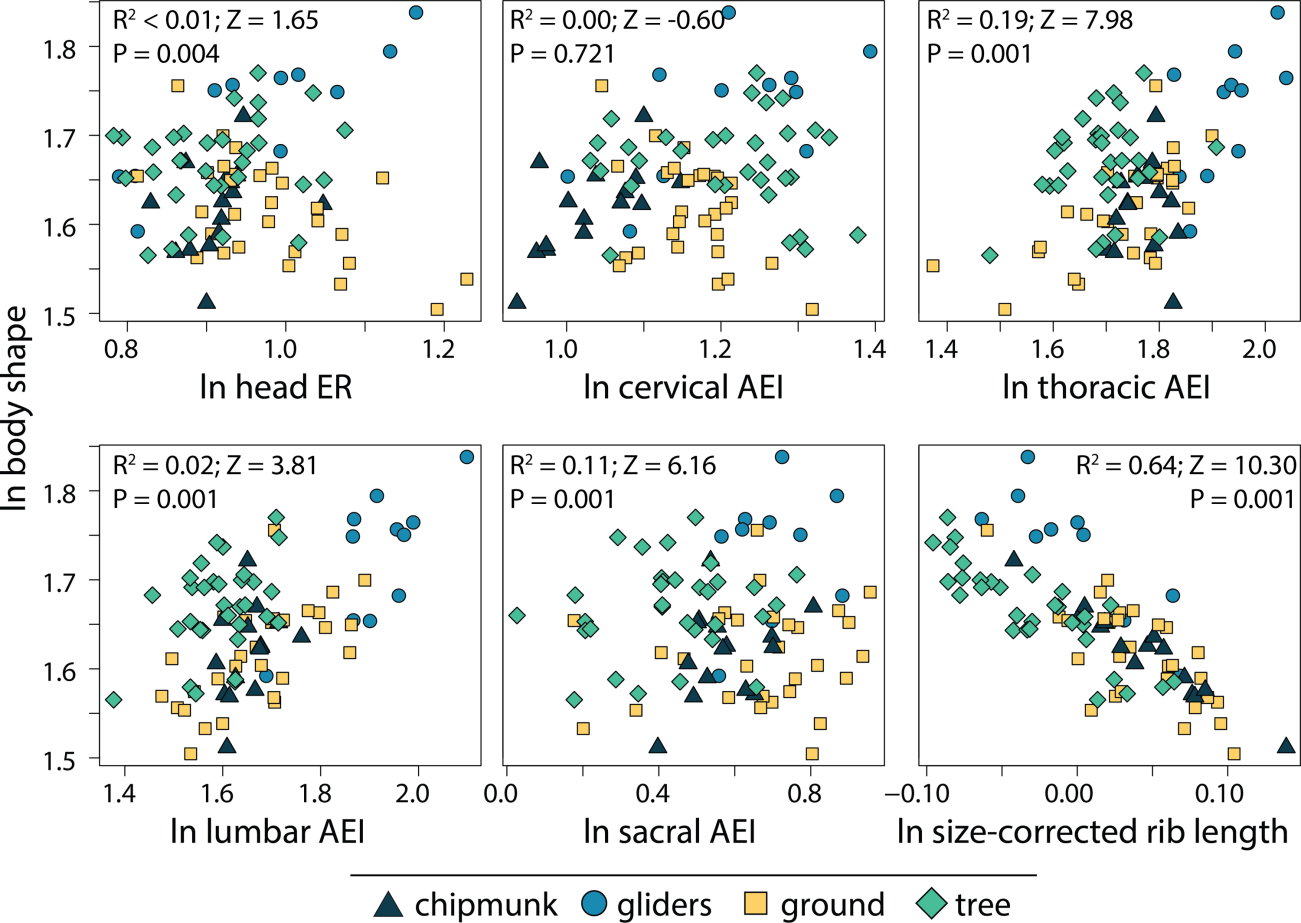
Scatterplots of ln body shape and morphological components underlying body shape. Body shape was quantified using the head-body elongation ratio. *R*^2^, Z scores, and p values were obtained from phylogenetic multiple regression with the random residual permutation procedure (RRPP). See Table 1 for full results.

We found that the relative length of the ribs (R^2^ = 0.64; P = 0.001) and elongation or shortening of the thoracic region (R^2^ = 0.19; P = 0.001) and sacral region (R^2^ = 0.11; P = 0.001) are most associated to body shape evolution across squirrels (Table 1; Fig. 7). Elongation or shortening of the head, cervical, and lumbar regions explained less than 2%. When examining each ecotype separately, we found that the relative length of the ribs was also the best explanatory variable of body shape in chipmunks (R^2^ = 0.48; P = 0.001), ground squirrels (R^2^, = 0.34; P = 0.001), and tree squirrels (R^2^ = 0.54; P = 0.001). In contrast, elongation or shortening of the head (R^2^ = 0.55; P = 0.003) was the best explanatory variable of body shape in gliding squirrels. The remaining morphological components explained 0 to 25% in body shape (Table 1; Fig. 7).

**Table 1:** Results of the phylogenetic multiple regression with the random residual permutation procedure (RRPP) to determine which morphological components contributed most to body shape evolution across all squirrels and within each ecotype. We adjusted all *P*-values using a Benjamini–Hochberg correction to reduce the type I error probability across multiple comparisons (Benjamini & Hochberg, 1995). Bold p-values indicate significance (*α* = 0.05). DF, degrees of freedom; SS, sum of squares.

	Morphological components	Df	SS	R^2^	F	Z	*P*
A. All ecotypes combined							
	ln head elongation ratio	1	0.00015	<0.01	4.280	1.650	**0.044**
	ln cervical AEI	1	0.00001	<0.01	0.150	−0.600	0.721
	ln thoracic AEI	1	0.01104	0.19	312.240	7.980	**0.001**
	ln lumbar AEI	1	0.00107	0.02	30.240	3.810	**0.001**
	ln sacral AEI	1	0.00620	0.11	175.300	6.160	**0.001**
	ln size-corrected rib length	1	0.03773	**0.64**	1066.640	10.300	**0.001**
	Residuals	80	0.00283	0.05			
	Total	86	0.05903				
B. Chipmunks							
	ln head elongation ratio	1	0.00214	0.08	167.980	5.210	**0.001**
	ln cervical AEI	1	0.00440	0.16	345.020	6.340	**0.001**
	ln thoracic AEI	1	0.00224	0.08	175.560	4.980	**0.001**
	ln lumbar AEI	1	0.00408	0.15	320.140	5.790	**0.001**
	ln sacral AEI	1	0.00110	0.04	86.530	4.320	**0.001**
	**ln size-corrected rib length**	1	0.01314	**0.48**	1031.090	8.310	**0.001**
	Residuals	8	0.00010	0.00			
	Total	14	0.02719				
C. Gliding squirrels							
	ln head elongation ratio	1	0.00200	**0.55**	52.220	3.240	**0.003**
	ln cervical AEI	1	0.00001	<0.01	0.300	−0.240	0.604
	ln thoracic AEI	1	0.00001	<0.01	0.350	−0.150	0.551
	ln lumbar AEI	1	0.00091	0.25	23.710	2.500	**0.004**
	ln sacral AEI	1	0.00018	0.05	4.600	1.320	0.084
	ln size-corrected rib length	1	0.00041	0.11	10.570	1.770	**0.031**
	Residuals	4	0.00015	0.04			
	Total	10	0.00367				
D. Ground squirrels							
	ln head elongation ratio	1	0.00397	0.24	60.070	4.590	**0.001**
	ln cervical AEI	1	0.00222	0.13	33.520	3.610	**0.001**
	ln thoracic AEI	1	0.00072	0.04	10.850	2.430	**0.006**
	ln lumbar AEI	1	0.00005	<0.01	0.680	0.210	0.428
	ln sacral AEI	1	0.00265	0.16	40.080	3.770	**0.001**
	**ln size-corrected rib length**	1	0.00575	**0.34**	86.920	5.060	**0.001**
	Residuals	22	0.00145	0.09			
	Total	28	0.01681				
E. Tree squirrels							
	ln head elongation ratio	1	0.00003	<0.01	1.440	0.780	0.23
	ln cervical AEI	1	0.00172	0.16	91.960	4.780	**0.001**
	ln thoracic AEI	1	0.00244	0.23	130.620	5.300	**0.001**
	ln lumbar AEI	1	0.00014	0.01	7.480	2.060	**0.013**
	ln sacral AEI	1	0.00006	0.01	3.310	1.380	0.082
	**ln size-corrected rib length**	1	0.00570	**0.54**	305.280	7.860	**0.001**
	Residuals	25	0.00047	0.04			
	Total	31	0.01055				

## Discussion

### Body shape allometry and limb length evolution

Although size is known to influence variation in cranial, vertebral, and appendicular shape within and across species (*e.g.*, Baliga & Mehta, 2016; Zelditch et al., 2017; Jones et al., 2018; Stepanova & Womack, 2020; Law et al., 2022), few studies have tested allometric patterns in overall body shapes (but see Friedman et al., 2019; Law, 2021b). Here, our results indicate that the relationship between body size and body shape is nuanced by ecological specialization; both body shape allometry and relationships between body shape and limb lengths differed between ecotypes (Fig. 3). Specifically, we found more elongate bodies with increasing size (*i.e.,* positive allometry) in chipmunk and gliding squirrel body shapes, more robust bodies with increasing size (*i.e.,* negative allometry) in ground squirrel body shapes, and no significant effect of body size on the evolution of tree squirrel body shapes.

As predicted, ground squirrels exhibited more robust bodies with increasing body size. This negative body shape allometry is consistent with what is found in terrestrial carnivorans (Law, Slater & Mehta, 2019; Law, 2021a). In large terrestrial mammals, robust body shapes (Law, 2021b) and low spinal flexibility (*i.e.,* dorsostability) of the vertebral column (Halpert, Jenkins Jr & Franks, 1987; Jones, 2015a) provide increased support against gravity for their heavier bodies (Kardong, 2014). For example, the lumbar vertebrae of large bovids and felids have more robust centra than found in smaller species, providing more stability against dorsoventral bending during running (Jones, 2015a). However, in Sciuridae, negative body shape allometry was only observed in ground squirrels, suggesting that fossoriality facilitated selection towards more robust bodies with increasing size.

Ground squirrels evolved relatively shorter forelimbs with increasing body elongation, reflecting the only significant trend observed between limb lengths and body shape in squirrels. The reduction or loss of limbs tend to evolve with body elongation in ectotherms (Gans, 1975; Wake, 1991; Wiens & Slingluff, 2001; Skinner, Lee & Hutchinson, 2008) and musteloid mammals (Law, Slater & Mehta, 2019). With the notable exception of snakes (Gans, 1975), this trend tends to be found in species that dig (Gans, 1975; Wake, 1991; Lee, 1998; Rieppel, 1998), spend significant time hunting in burrows (Law, Slater & Mehta, 2019), or shelter in leaf litter and other surface debris (Gans, 1975). Additionally, elongate, limb-reduced bodies tend to be associated with a small body size (Lee, 1998; Rieppel, 1998). These trends are only corroborated by our results for ground squirrels, the most fossorial of the four ecotypes. This allometric trend may suggest that small, elongate ground squirrels rely more on their relatively shorter forelimbs to provide the necessary support, increased mechanical advantage, and force production (Lagaria & Youlatos, 2006; Samuels & Van Valkenburgh, 2008) needed to dig large burrow systems (Goldstein, 1972; Casinos, Quintana & Viladiu, 1993) compared to larger, more robust ground squirrels. Additionally, we found reduction of forelimb lengths in elongate species but not hind limb lengths, as seen in both mustelids (Law, Slater & Mehta, 2019) and ectotherms (Gans, 1975; Wiens & Slingluff, 2001; Brandley, Huelsenbeck & Wiens, 2008; Morinaga & Bergmann, 2020). Our research marked the first evidence of this trend in rodents.

In contrast to ground squirrels and terrestrial carnivorans, gliding squirrels exhibited positive body shape allometry, in which larger species exhibit more elongate bodies. Additionally, we confirm Thorington & Heaney’s (1981) hypothesis that small tree and gliding squirrels exhibit similarly robust bodies, while large gliding squirrels are more elongate than large tree squirrels. An elongate body could enable more aerodynamic and maneuverable gliding in large gliding squirrels, which face increased effects of gravity and drag due to their larger sizes. Because body mass is expected to increase proportionally to the cube of linear measurements whereas wing area increases proportionally to the square, wing loading (body mass/patagium area) would naturally be higher in large gliding squirrels (Thorington & Heaney, 1981). There are multiple pathways through which large gliding squirrels can compensate for this, including body elongation allometry (*i.e.,* decreasing relative body weight with size) and limb length allometry (*i.e.,* disproportionately increasing patagium area with size). The elongate forms of large gliding squirrels correspond to a 16% lower relative body weight than small gliding squirrels, decreasing the effects of wing loading on large squirrels (Thorington & Heaney, 1981). Interestingly, relative limb length did not increase with body elongation (Fig. 5), possibly due to constraints from scansorial and gliding locomotion on forelimb length. Exceedingly long limbs could interfere with the squirrels’ ability to climb, reduce maneuverability when gliding, and risk more frequent breakage. Despite the lack of relationship between limb length and body elongation, gliding squirrels increase the area of their patagium through the styliform cartilage on their wrists (Johnson-Murray, 1977; Thorington, Darrow & Anderson, 1998). The styliform extension, while isometric with respect to size (Thorington & Heaney, 1981), could reduce selection towards positive allometry in limb length by both increasing maneuverability (acting similarly to a winglet on an airplane wing) and reducing wing loading (Thorington, Darrow & Anderson, 1998).

Despite the lack of relative limb length allometry, we confirm that gliding squirrels exhibited relatively longer forelimbs than all other ecotypes (Fig. 6; Peterka, 1936; Bryant, 1945; Thorington & Heaney, 1981), a pattern that has been found across all gliding mammals (Grossnickle et al., 2020). Therefore, the elongate bodies and relatively longer forelimbs together in gliding squirrels could reduce the effects of wing loading on the gliding capabilities of larger species. Interestingly, the observation that gliders exhibited relatively longer hind limbs compared to the remaining ecotypes was not as well supported statistically (Fig. 6). A possible explanation for this pattern is that, because of the positioning of the forelimbs in the patagium, elongating the forelimbs may better support a wider plagiopatagium than elongating the hind limbs. Longer hind limbs would increase the uropatagium area and thus decrease the aspect ratio (wingspan^2^/wing area) of gliding squirrels, which is already quite low compared to birds (Thorington & Heaney, 1981). While a low aspect ratio could facilitate landing (Zimmerman, 1932; Thorington & Heaney, 1981) and increase maneuverability (Norberg, 1995), Thorington & Heaney (1981) proposed that this low aspect ratio could decrease glide ratio (horizontal distance/altitude loss). A low glide ratio could negatively impact gliding ability, decreasing the selective pressure for long hind limb length in gliding squirrels. Additionally, the relative hind limb lengths of gliding squirrels increased with body elongation when the metatarsals were included. The patagium connects at the ankle, so large gliders’ higher metatarsal length does not affect wing area despite uropatagium area being relatively larger in large gliding squirrels, possibly as an adaptation to compensate for their high wing loading (Thorington & Heaney, 1981). This finding could instead be related to the start of gliding locomotion; larger, more elongate gliding squirrels may require more momentum from long metatarsals to propel themselves off of branches. Furthermore, aspects of the morphology other than forelimb and hind limb length, such as patagium musculature (Johnson-Murray, 1977), could be altered to compensate for wing loading or increase maneuverability in large species.

Generalist chipmunks did not exhibit similar trends to fossorial ground squirrels; chipmunks showed neither a negative body shape allometric trend nor negative trend between relative limb length and body elongation. A possible explanation is that chipmunks exhibit a narrower range of body sizes, from the 32–50 g least chipmunk to the 66–150 g Eastern chipmunk (Kurta, 1995; Reid, 2006; Roland, 2009). Chipmunks are also small compared to ground squirrels, which range from the 96–117 g white-tailed antelope squirrel to the 8 kg Olympic marmot. Chipmunks and small ground squirrels tend to burrow for shelter, whereas larger ground squirrels and marmots tend to build extensive burrow systems (Armstrong, 2007). Therefore, these differences in size-related burrowing behavior and lack of body size diversity within chipmunks could explain their different trends compared to ground squirrels. Instead, chipmunks exhibit more elongate bodies with increasing body size, an adaptation that may provide larger chipmunk species increased maneuverability when climbing trees and navigating tight tree hollows.

Lastly, there was no relationship between body size and body shape in tree squirrels. An elongate body could be relatively unimportant compared to relative tail length for arboreal maneuvering. Tree squirrels exhibit relatively longer tails than ground squirrels (Hayssen, 2008), which may facilitate balance during arboreal locomotion (Buck, Tolman & Tolman, 1925; Siegel, 1970; Hayssen, 2008) and help right falling squirrels (Fukushima et al., 2021). The relatively longer tails of larger squirrels could contribute to large tree squirrels’ ability to navigate arboreal terrain despite the lack of body shape allometry.

### Allometry of axial skeleton components

Locomotion can impact the evolution of the axial skeleton, especially the thoracolumbar region, which is the main axial region responsible for generating the propulsive forces necessary for locomotion (Boszczyk, Boszczyk & Putz, 2001; Kardong, 2014). Suspensory mammals with dorsostabile adaptations have higher variability in presacral vertebral number than running mammals with dorsomobile (high spinal flexibility) adaptations (Williams et al., 2019). As size increases, the energetic and biomechanical costs of dorsomobile running also increase. Therefore, a trade-off between stabilization and efficiency may have led to increased lumbar stability with increasing body size in large running mammals such as bovids (Jones, 2015a). Here, we found that allometry influenced the evolution of the thoracolumbar region in only the ground and gliding squirrels. Unsurprisingly, ground squirrels evolved a more robust thoracic region and relatively longer ribs with increasing body size. A more robust thoracic region and rib cage contributes to dorsostability in large squirrels (Boszczyk, Boszczyk & Putz, 2001; Jones, 2015a; Jones, 2015b), which could support their bodies as they dig large tunnel systems. In contrast, gliding squirrels evolved a more elongate lumbar region. The increased elongation in the lumbar region of larger gliding squirrels could be an adaptation to provide dorsoventral maneuverability and flexibility (Boszczyk, Boszczyk & Putz, 2001; Jones, 2015a; Jones, 2015b) while gliding.

The cervical region was the only morphological component measured that consistently exhibited positive allometry across all four ecotypes. Increased elongation of the cervical region with increasing size is counterintuitive in ground squirrels, since robust cervical vertebrae could provide more support for the head as body size increases. In fact, across mammals, the cervical spine tends to shorten relatively with increasing body size, albeit these allometric trends differ between different clades (Arnold, Amson & Fischer, 2017). A possible explanation for the positive cervical allometry in squirrels is that the flexibility provided by a relatively longer neck is advantageous for large squirrels regardless of ecotype, allowing more maneuverability of their necks when navigating complex terrain or burrowing. Elongation of other vertebral regions contributes to dorsal flexibility (Boszczyk, Boszczyk & Putz, 2001; Jones, 2015a; Jones, 2015b), and elongation of the cervical region may similarly facilitate locomotion. Additionally, it is possible that an ability to see a large range of land could be advantageous for large ground squirrels. To avoid predation, large ground squirrels must seek out abundant piles of boulders and rocks for cover, while smaller ground squirrels tend to use vegetation for cover (Armstrong, 2007). Larger ground squirrels’ need to scan the landscape to look for large boulders could explain selective pressures for more flexible necks. Furthermore, ground squirrels exhibit bipedal posture when threatened by potential predators (Swaisgood, Rowe & Owings, 1999), and an elongate neck could better enhance the view of their environment that this posture provides, especially for large, conspicuous squirrels. Meanwhile, elongation of the cervical vertebrae could be advantageous for gliding squirrels as it could contribute to the evolution of a more elongate and aerodynamic form.

Gliding and ground squirrels exhibited a positive allometric trend for head elongation ratio. More elongate heads in larger gliders may decrease drag and make gliding more aerodynamic. The positive allometric trend in ground squirrels, however, is more surprising. Some large ground squirrel species use their heads to push stones aside when burrowing (Kwiecinski, 1998), which a more robust head would better facilitate. The positive allometric trends of head elongation ratio in all ecotypes follow similar trends of craniofacial evolutionary allometry (CREA), the tendency for larger species to evolve relatively longer rostrums. CREA has been observed in a diverse range of mammalian clades, including antelopes, kangaroos, bats, and mongooses (Cardini & Polly, 2013; Cardini et al., 2015; Arbour, Curtis & Santana, 2021). CREA was also observed in tree squirrels of the subfamily Sciurinae (Cardini & Polly, 2013), suggesting that the elongation of the rostrum rather than the braincase contributed to the overall elongation of the cranium in squirrels.

### Morphological components of body shape

The relative depth of the ribcage (R^2^ = 0.64) and thoracic vertebrae (R^2^ = 0.19) exhibited the strongest relationships with body shape in squirrels, together contributing to 83% of total body shape. These structures are vital to supporting the body against gravity and supporting the limbs during propulsive forces (Kardong, 2014). Our findings slightly differ from trends described in carnivorans, where elongation of the lumbar region (R^2^ = 0.41) followed by relative length of the ribs (R^2^ = 0.21) and elongation of the thoracic vertebrae (R^2^ = 0.14) exhibited the strongest relationships with body shape (Law, 2019). The dorsoventral flexibility that an elongate lumbar region provides (Boszczyk, Boszczyk & Putz, 2001; Jones, 2015a; Jones, 2015b) could allow for increased maneuverability when hunting, especially during pouncing and chasing behaviors. This could explain why the lumbar region is associated with body shape far more in carnivorans compared to squirrels.

We also found that, consistent with findings in other taxonomic groups, the morphological components best associated with body shape evolution differ between ecotype or clade. In squirrels, relative rib lengths explained the most variation out of any morphological component in most ecotypes (ground squirrels R^2^ = 0.34; chipmunks R^2^ = 0.48; tree squirrels R^2^ = 0.54) except for gliding squirrels, where head elongation (R^2^ = 0.55) explained the majority of body shape evolution. Across carnivoran families, elongation or shortening of the head, cervical, and/or lumbar regions, as well as the relative length of the ribs explained the most variation in body shape (Law, Slater & Mehta, 2019; Law, 2021b). Even carnivorans that exhibit incomplete convergence towards body elongation (*i.e.,* weasels, civets, and mongooses) display multiple pathways towards their converging elongate body plans (Law, 2022). Altogether, these results demonstrate how the evolution of different body shapes can arise through multiple diverging evolutionary pathways (Ward & Mehta, 2010; Ward & Mehta, 2014; Morinaga & Bergmann, 2017; Bergmann & Morinaga, 2019; Law, 2021b; Law, 2022).

## Conclusion

Habitat use influenced allometric patterns in body shape and its underlying morphological components in Sciuridae. First, ground squirrels exhibit negative body shape allometry, while gliding squirrels and chipmunks evolved more elongate bodies with increasing size. Second, only ground squirrels exhibit a relationship between the forelimb and body shape, where more elongate species exhibit relatively shorter forelimbs. Finally, the relative length of the ribs and elongation or shortening of the thoracic region explained the most of body shape evolution across squirrels. Altogether, because body shape evolution has far-reaching effects on the physiology, biomechanics, and ecology of species, including heat conservation (Brown & Lasiewski, 1972), locomotion (Sharpe et al., 2015; Ward et al., 2015), and ability to exploit niches (Law, 2019), these results provide a strong morphological foundation for future research investigating the evo-devo and evolutionary ecology of squirrel and other mammalian body plans.

##  Supplemental Information

10.7717/peerj.14800/supp-1 Supplemental Information 1 Supplementary Tables

10.7717/peerj.14800/supp-2 Supplemental Information 2 HbER data for R scripts

10.7717/peerj.14800/supp-3 Supplemental Information 3 Raw data used to calculate hbER

10.7717/peerj.14800/supp-4 Supplemental Information 4 Squirrel phylogeny for analyses

10.7717/peerj.14800/supp-5 Supplemental Information 5 R script for PGLS and ANCOVA analyses on limb lengths

10.7717/peerj.14800/supp-6 Supplemental Information 6 R script for multiple regression for body shape components

10.7717/peerj.14800/supp-7 Supplemental Information 7 R script for PGLS and ANCOVA for body shape analyses

## Additional Information and Declarations

### Data Availability

The following information was supplied regarding data availability: Raw data of body shape and body shape components are available in the Supplemental Files.
