# Supplementary material for: Scaling patterns of body plans differ among squirrel ecotypes
Source: PeerJ. 2023 Jan 25;11:e14800. doi: 10.7717/peerj.14800 (PMC9884040; doi:10.7717/peerj.14800)
Supplement: Supplemental Information 1 [file peerj-11-14800-s001.docx]

Supplementary Materials for *Scaling patterns of body plans differ among squirrel ecotypes*

Tate J. Linden^1^, Abigail E. Burtner^1^, Johannah Rickman^1^, Annika McFeely^1^, Sharlene E. Santana^1^, Chris J. Law^1,2,3*^

**Supplementary Tables**

Table S1. Catalog numbers of specimens used

Table S2. Output tables of PGLS regressions between body shape and body size and between body shape components and body size.

Table S3. Slope and intercept coefficients of PGLS models between body shape components, size, and ecotype.

Table S4. Output tables of PGLS regressions between size-corrected limb lengths and body shape.

Table S5. Slope and intercept coefficients of PGLS models between limb length and body shape.

**Table S1**. Catalog numbers of specimens used. KU = Biodiversity Institute and Natural History Museum; LACM = Natural History Museum of Los Angeles County; MCZ = Museum of Comparative Zoology; MVZ = Museum of Vertebrate Zoology; PSMP = University of Puget Sound Museum; UBC = Beaty Museum; UF = Florida Museum of Natural History; USNM = National Museum of Natural History; UWBM = Burke Museum of Natural History and Culture

| species | sex | ecotype | catalog |
| --- | --- | --- | --- |
| Tamias_alpinus | F | chipmunk | MVZ207209 |
| Tamias_alpinus | F | chipmunk | MVZ207210 |
| Tamias_amoenus | F | chipmunk | UWBM31698 |
| Tamias_amoenus | M | chipmunk | UWBM35963 |
| Tamias_amoenus | F | chipmunk | UWBM39189 |
| Tamias_amoenus | F | chipmunk | UWBM75475 |
| Tamias_amoenus | F | chipmunk | UWBM78184 |
| Tamias_cinereicollis | F | chipmunk | UWBM38257 |
| Tamias_dorsalis | M | chipmunk | UWBM78903 |
| Tamias_dorsalis | F | chipmunk | UWBM78908 |
| Tamias_dorsalis | M | chipmunk | UWBM79657 |
| Tamias_merriami | M | chipmunk | UWBM60172 |
| Tamias_merriami | F | chipmunk | UWBM60174 |
| Tamias_minimus | F | chipmunk | MVZ219925 |
| Tamias_minimus | M | chipmunk | MVZ219926 |
| Tamias_minimus | M | chipmunk | UWBM30813 |
| Tamias_minimus | M | chipmunk | UWBM77677 |
| Tamias_minimus | F | chipmunk | UWBM77683 |
| Tamias_panamintinus | F | chipmunk | MVZ224274 |
| Tamias_panamintinus | M | chipmunk | MVZ224275 |
| Tamias_quadrivittatus | F | chipmunk | UWBM35298 |
| Tamias_quadrivittatus | F | chipmunk | UWBM35298 |
| Tamias_quadrivittatus | F | chipmunk | UWBM35299 |
| Tamias_quadrivittatus | F | chipmunk | UWBM35299 |
| Tamias_quadrivittatus | M | chipmunk | UWBM35301 |
| Tamias_quadrivittatus | F | chipmunk | UWBM36479 |
| Tamias_quadrivittatus | F | chipmunk | UWBM36480 |
| Tamias_ruficaudus | F | chipmunk | UWBM34354 |
| Tamias_ruficaudus | M | chipmunk | UWBM34355 |
| Tamias_ruficaudus | M | chipmunk | UWBM34357 |
| Tamias_ruficaudus | M | chipmunk | UWBM34357 |
| Tamias_ruficaudus | M | chipmunk | UWBM34360 |
| Tamias_ruficaudus | M | chipmunk | UWBM76595 |
| Tamias_senex | F | chipmunk | UWBM78783 |
| Tamias_sibiricus | F | chipmunk | UWBM39255 |
| Tamias_sibiricus | F | chipmunk | UWBM39255 |
| Tamias_sibiricus | F | chipmunk | UWBM39257 |
| Tamias_sibiricus | M | chipmunk | UWBM39258 |
| Tamias_sibiricus | F | chipmunk | UWBM39259 |
| Tamias_sibiricus | M | chipmunk | UWBM77286 |
| Tamias_siskiyou | U | chipmunk | UWBM80827 |
| Tamias_siskiyou | F | chipmunk | UWBM80828 |
| Tamias_siskiyou | F | chipmunk | UWBM80829 |
| Tamias_speciosus | F | chipmunk | UWBM43059 |
| Tamias_speciosus | F | chipmunk | UWBM60295 |
| Tamias_speciosus | F | chipmunk | UWBM60297 |
| Tamias_speciosus | M | chipmunk | UWBM60299 |
| Tamias_striatus | M | chipmunk | UWBM35246 |
| Tamias_townsendii | F | chipmunk | UWBM35167 |
| Tamias_townsendii | F | chipmunk | UWBM35180 |
| Tamias_townsendii | F | chipmunk | UWBM35182 |
| Tamias_townsendii | F | chipmunk | UWBM35208 |
| Tamias_townsendii | F | chipmunk | UWBM35213 |
| Aeretes_melanopterus | U | gliding | MCZ19994 |
| Eoglaucomys_fimbriatus | F | gliding | USNM173363 |
| Eoglaucomys_fimbriatus | F | gliding | USNM173365 |
| Eupetaurus_cinereus | M | gliding | UF28583 |
| Glaucomys_sabrinus | M | gliding | UWBM35051 |
| Glaucomys_sabrinus | F | gliding | UWBM35053 |
| Glaucomys_sabrinus | F | gliding | UWBM35056 |
| Glaucomys_sabrinus | F | gliding | UWBM35058 |
| Glaucomys_sabrinus | M | gliding | UWBM35077 |
| Glaucomys_volans | M | gliding | MVZ97094 |
| Glaucomys_volans | F | gliding | UWBM35225 |
| Glaucomys_volans | F | gliding | UWBM43897 |
| Iomys_horsfieldii | F | gliding | PSM10370 |
| Petaurista_alborufus | F | gliding | MVZ174855 |
| Petaurista_alborufus | U | gliding | MVZ183717 |
| Petaurista_elegans | U | gliding | MCZ36579 |
| Petaurista_elegans | U | gliding | MCZ36580 |
| Petaurista_petaurista | F | gliding | USNM197320 |
| Petaurista_petaurista | M | gliding | USNM588884 |
| Petaurista_philippensis | M | gliding | USNM314973 |
| Pteromys_volans | M | gliding | UWBM39689 |
| Ammospermophilus_leucurus | F | ground | MVZ216222 |
| Ammospermophilus_leucurus | F | ground | UWBM74639 |
| Ammospermophilus_leucurus | F | ground | UWBM74641 |
| Ammospermophilus_leucurus | F | ground | UWBM74643 |
| Ammospermophilus_leucurus | M | ground | UWBM74646 |
| Ammospermophilus_leucurus | M | ground | UWBM74647 |
| Ammospermophilus_nelsoni | F | ground | MVZ234368 |
| Callospermophilus_lateralis | M | ground | UWBM38959 |
| Callospermophilus_lateralis | F | ground | UWBM38960 |
| Callospermophilus_lateralis | F | ground | UWBM38961 |
| Callospermophilus_lateralis | F | ground | UWBM38964 |
| Callospermophilus_lateralis | F | ground | UWBM38965 |
| Callospermophilus_madrensis | M | ground | MVZ99799 |
| Callospermophilus_saturatus | M | ground | UWBM31137 |
| Callospermophilus_saturatus | M | ground | UWBM38984 |
| Callospermophilus_saturatus | F | ground | UWBM44578 |
| Callospermophilus_saturatus | F | ground | UWBM44586 |
| Cynomys_gunnisoni | M | ground | MVZ99763 |
| Cynomys_ludovicianus | M | ground | UWBM75774 |
| Cynomys_mexicanus | M | ground | MVZ91189 |
| Ictidomys_mexicanus | M | ground | MVZ93788 |
| Ictidomys_mexicanus | F | ground | MVZ93789 |
| Marmota_broweri | M | ground | UWBM32251 |
| Marmota_broweri | M | ground | UWBM82831 |
| Marmota_caligata | F | ground | UWBM13561 |
| Marmota_caligata | F | ground | UWBM31095 |
| Marmota_caligata | M | ground | UWBM35529 |
| Marmota_caligata | M | ground | UWBM35997 |
| Marmota_caligata | U | ground | UWBM43431 |
| Marmota_flaviventris | F | ground | UWBM32500 |
| Marmota_flaviventris | M | ground | UWBM77395 |
| Marmota_flaviventris | M | ground | UWBM82825 |
| Marmota_monax | F | ground | UWBM38327 |
| Marmota_monax | M | ground | UWBM39792 |
| Marmota_monax | F | ground | UWBM82380 |
| Marmota_olympus | F | ground | PSMP2520 |
| Marmota_vancouverensis | M | ground | UBC019561 |
| Marmota_vancouverensis | M | ground | UBC019586 |
| Otospermophilus_beecheyi | U | ground | UWBM15430 |
| Otospermophilus_beecheyi | M | ground | UWBM37043 |
| Otospermophilus_beecheyi | M | ground | UWBM39380 |
| Otospermophilus_beecheyi | F | ground | UWBM80593 |
| Otospermophilus_variegatus | M | ground | UWBM79876 |
| Poliocitellus_franklinii | F | ground | UWBM33260 |
| Poliocitellus_franklinii | F | ground | UWBM33261 |
| Urocitellus_armatus | F | ground | MVZ64641 |
| Urocitellus_armatus | F | ground | MVZ72124 |
| Urocitellus_beldingi | F | ground | UWBM42455 |
| Urocitellus_beldingi | F | ground | UWBM42456 |
| Urocitellus_beldingi | U | ground | UWBM42457 |
| Urocitellus_beldingi | F | ground | UWBM42458 |
| Urocitellus_beldingi | U | ground | UWBM42459 |
| Urocitellus_beldingi | F | ground | UWBM80581 |
| Urocitellus_columbianus | F | ground | UWBM12600 |
| Urocitellus_columbianus | M | ground | UWBM14267 |
| Urocitellus_columbianus | F | ground | UWBM37533 |
| Urocitellus_columbianus | F | ground | UWBM74770 |
| Urocitellus_columbianus | M | ground | UWBM80405 |
| Urocitellus_elegans | M | ground | UWBM33286 |
| Urocitellus_parryii | F | ground | UWBM39263 |
| Urocitellus_parryii | F | ground | UWBM39266 |
| Urocitellus_parryii | F | ground | UWBM39268 |
| Urocitellus_parryii | F | ground | UWBM39270 |
| Urocitellus_parryii | M | ground | UWBM39271 |
| Urocitellus_parryii | F | ground | UWBM39623 |
| Urocitellus_richardsonii | M | ground | UWBM32960 |
| Urocitellus_richardsonii | M | ground | UWBM32961 |
| Urocitellus_richardsonii | M | ground | UWBM32962 |
| Urocitellus_townsendii | U | ground | UWBM28071 |
| Urocitellus_townsendii | F | ground | UWBM78314 |
| Urocitellus_townsendii | F | ground | UWBM78333 |
| Xerospermophilus_spilosoma | F | ground | UWBM35279 |
| Xerospermophilus_spilosoma | F | ground | UWBM35282 |
| Xerospermophilus_spilosoma | M | ground | UWBM35284 |
| Xerospermophilus_spilosoma | M | ground | UWBM35285 |
| Xerospermophilus_spilosoma | M | ground | UWBM35286 |
| Xerospermophilus_tereticaudus | F | ground | UWBM35502 |
| Xerus_erythropus | M | ground | MVZ115437 |
| Xerus_inauris | F | ground | MVZ117287 |
| Callosciurus_erythraeus | F | tree | CAS6370 |
| Callosciurus_erythraeus | F | tree | CAS6371 |
| Callosciurus_prevostii | M | tree | LACM097638 |
| Callosciurus_prevostii | F | tree | LACM97346 |
| Callosciurus_pygerythrus | F | tree | PSM16608 |
| Callosciurus_pygerythrus | M | tree | PSM27419 |
| Funambulus_palmarum | F | tree | MVZ183704 |
| Funambulus_palmarum | M | tree | MVZ183706 |
| Funambulus_pennantii | F | tree | UF28826 |
| Funambulus_pennantii | M | tree | UF30290 |
| Funisciurus_pyrropus | F | tree | MVZ196233 |
| Heliosciurus_mutabilis | M | tree | MVZ220919 |
| Heliosciurus_rufobrachium | U | tree | MCZ35324 |
| Heliosciurus_rufobrachium | U | tree | MCZ35327 |
| Heliosciurus_rufobrachium | M | tree | MVZ196227 |
| Microsciurus_flaviventer | M | tree | MVZ124046 |
| Microsciurus_flaviventer | M | tree | MVZ154935 |
| Microsciurus_flaviventer | F | tree | MVZ154936 |
| Paraxerus_boehmi | F | tree | LSUMZ37843 |
| Prosciurillus_murinus | M | tree | LSUMZ38363 |
| Protoxerus_stangeri | F | tree | MVZ196228 |
| Protoxerus_stangeri | M | tree | MVZ196230 |
| Ratufa_bicolor | M | tree | MVZ123699 |
| Ratufa_macroura | U | tree | UWBM21183 |
| Sciurus_aberti | M | tree | UWBM35277 |
| Sciurus_aberti | F | tree | UWBM35278 |
| Sciurus_aberti | F | tree | UWBM74145 |
| Sciurus_aestuans | U | tree | MVZ182069 |
| Sciurus_aestuans | F | tree | MVZ182070 |
| Sciurus_aureogaster | F | tree | LACM053653 |
| Sciurus_aureogaster | F | tree | LACM053654 |
| Sciurus_carolinensis | F | tree | UWBM35488 |
| Sciurus_carolinensis | M | tree | UWBM39373 |
| Sciurus_carolinensis | M | tree | UWBM39787 |
| Sciurus_carolinensis | F | tree | UWBM43904 |
| Sciurus_carolinensis | F | tree | UWBM75810 |
| Sciurus_colliaei | F | tree | LACM058790 |
| Sciurus_deppei | M | tree | MVZ98319 |
| Sciurus_deppei | F | tree | MVZ98326 |
| Sciurus_griseus | F | tree | UWBM35487 |
| Sciurus_griseus | M | tree | UWBM42330 |
| Sciurus_griseus | F | tree | UWBM74097 |
| Sciurus_griseus | F | tree | UWBM76257 |
| Sciurus_griseus | F | tree | UWBM76264 |
| Sciurus_nayaritensis | M | tree | MVZ109632 |
| Sciurus_niger | M | tree | UWBM35226 |
| Sciurus_niger | F | tree | UWBM35229 |
| Sciurus_niger | F | tree | UWBM35230 |
| Sciurus_niger | M | tree | UWBM35231 |
| Sciurus_niger | M | tree | UWBM81529 |
| Sciurus_spadiceus | U | tree | MVZ166023 |
| Sciurus_stramineus | M | tree | MVZ135638 |
| Sciurus_variegatoides | M | tree | MVZ131091 |
| Sciurus_variegatoides | F | tree | MVZ131095 |
| Sciurus_vulgaris | M | tree | UWBM39069 |
| Sciurus_vulgaris | F | tree | UWBM39260 |
| Sciurus_vulgaris | F | tree | UWBM77295 |
| Sundasciurus_juvencus | U | tree | KU165382 |
| Tamiasciurus_douglasii | F | tree | UWBM39137 |
| Tamiasciurus_douglasii | F | tree | UWBM44366 |
| Tamiasciurus_douglasii | F | tree | UWBM81947 |
| Tamiasciurus_douglasii | F | tree | UWBM82060 |
| Tamiasciurus_douglasii | F | tree | UWBM82088 |
| Tamiasciurus_hudsonicus | M | tree | UWBM35237 |
| Tamiasciurus_hudsonicus | F | tree | UWBM81502 |
| Tamiasciurus_hudsonicus | M | tree | UWBM81510 |
| Tamiasciurus_hudsonicus | M | tree | UWBM81909 |
| Tamiasciurus_hudsonicus | F | tree | UWBM82174 |
| Tamiops_maritimus | M | tree | MVZ186481 |
| Tamiops_swinhoei | F | tree | UWBM75283 |

**Table S2.** Output tables of PGLS regressions between body shape and body size and between body shape components and body size.

| body shape ~ body size | |  |  |  |  |  |  |
| --- | --- | --- | --- | --- | --- | --- | --- |
|  |  | Estimate | StdErr | t.value | lowerbootCI | upperbootCI | p.value |
|  | intercept | 1.731 | 0.088 | 19.587 | 1.552 | 1.903 | 0.000 |
|  | ln body size | -0.023 | 0.023 | -1.013 | -0.067 | 0.023 | 0.314 |
|  |  |  |  |  |  |  |  |
| body shape ~ body size*ecotype | | |  |  |  |  |  |
|  |  | Estimate | StdErr | t.value | lowerbootCI | upperbootCI | p.value |
|  | intercept | 0.904 | 0.339 | 2.664 | 0.237 | 1.555 | 0.009 |
|  | ln body size | 0.237 | 0.112 | 2.107 | 0.021 | 0.460 | 0.038 |
|  | gliding | 0.369 | 0.364 | 1.013 | -0.313 | 1.075 | 0.314 |
|  | ground | 1.101 | 0.351 | 3.137 | 0.414 | 1.800 | 0.002 |
|  | tree | 0.838 | 0.354 | 2.369 | 0.170 | 1.539 | 0.020 |
|  | ln body size * gliding | -0.114 | 0.118 | -0.964 | -0.344 | 0.114 | 0.338 |
|  | ln body size * ground | -0.343 | 0.115 | -2.983 | -0.573 | -0.114 | 0.004 |
|  | ln body size * tree | -0.257 | 0.116 | -2.221 | -0.487 | -0.039 | 0.029 |
|  |  |  |  |  |  |  |  |
| headER ~ body size | |  |  |  |  |  |  |
|  |  | Estimate | StdErr | t.value | lowerbootCI | upperbootCI | p.value |
|  | intercept | 0.306 | 0.099 | 3.090 | 0.115 | 0.499 | 0.003 |
|  | ln body size | 0.176 | 0.026 | 6.764 | 0.124 | 0.225 | 0.000 |
|  |  |  |  |  |  |  |  |
| headER ~ body size*ecotype | | |  |  |  |  |  |
|  |  | Estimate | StdErr | t.value | lowerbootCI | upperbootCI | p.value |
|  | intercept | 0.269 | 0.470 | 0.573 | -0.610 | 1.177 | 0.568 |
|  | ln body size | 0.214 | 0.155 | 1.376 | -0.087 | 0.507 | 0.173 |
|  | gliding | -0.079 | 0.504 | -0.157 | -1.026 | 0.866 | 0.875 |
|  | ground | -0.101 | 0.486 | -0.208 | -0.965 | 0.824 | 0.836 |
|  | tree | 0.430 | 0.490 | 0.878 | -0.531 | 1.402 | 0.383 |
|  | ln body size * gliding | -0.004 | 0.163 | -0.023 | -0.313 | 0.306 | 0.981 |
|  | ln body size * ground | 0.008 | 0.159 | 0.050 | -0.298 | 0.301 | 0.960 |
|  | ln body size * tree | -0.154 | 0.160 | -0.958 | -0.470 | 0.159 | 0.341 |
|  |  |  |  |  |  |  |  |
| cervical AEI ~ body size | |  |  |  |  |  |  |
|  |  | Estimate | StdErr | t.value | lowerbootCI | upperbootCI | p.value |
|  | intercept | 0.402 | 0.086 | 4.660 | 0.234 | 0.564 | 0.000 |
|  | ln body size | 0.221 | 0.022 | 10.053 | 0.181 | 0.267 | 0.000 |
|  |  |  |  |  |  |  |  |
| cervical AEI ~ body size*ecotype | | |  |  |  |  |  |
|  |  | Estimate | StdErr | t.value | lowerbootCI | upperbootCI | p.value |
|  | intercept | -0.454 | 0.299 | -1.521 | -1.016 | 0.107 | 0.132 |
|  | ln body size | 0.495 | 0.097 | 5.129 | 0.313 | 0.666 | 0.000 |
|  | gliding | 0.599 | 0.339 | 1.765 | -0.072 | 1.222 | 0.081 |
|  | ground | 1.149 | 0.316 | 3.640 | 0.544 | 1.723 | 0.000 |
|  | tree | 0.683 | 0.323 | 2.116 | 0.066 | 1.255 | 0.037 |
|  | ln body size * gliding | -0.208 | 0.106 | -1.968 | -0.404 | -0.001 | 0.053 |
|  | ln body size * ground | -0.370 | 0.101 | -3.679 | -0.559 | -0.178 | 0.000 |
|  | ln body size * tree | -0.221 | 0.103 | -2.152 | -0.404 | -0.019 | 0.034 |
|  |  |  |  |  |  |  |  |
| thoracic AEI ~ body size | |  |  |  |  |  |  |
|  |  | Estimate | StdErr | t.value | lowerbootCI | upperbootCI | p.value |
|  | intercept | 1.942 | 0.140 | 13.871 | 1.649 | 2.223 | 0.000 |
|  | ln body size | -0.063 | 0.036 | -1.750 | -0.134 | 0.011 | 0.084 |
|  |  |  |  |  |  |  |  |
| thoracic AEI ~ body size*ecotype | | |  |  |  |  |  |
|  |  | Estimate | StdErr | t.value | lowerbootCI | upperbootCI | p.value |
|  | intercept | 1.725 | 0.499 | 3.455 | 0.828 | 2.695 | 0.001 |
|  | ln body size | 0.006 | 0.162 | 0.036 | -0.300 | 0.301 | 0.972 |
|  | gliding | -0.261 | 0.562 | -0.464 | -1.335 | 0.757 | 0.644 |
|  | ground | 0.540 | 0.526 | 1.028 | -0.490 | 1.482 | 0.307 |
|  | tree | -0.069 | 0.538 | -0.128 | -1.095 | 0.909 | 0.898 |
|  | ln body size * gliding | 0.117 | 0.176 | 0.664 | -0.206 | 0.451 | 0.509 |
|  | ln body size * ground | -0.175 | 0.168 | -1.040 | -0.474 | 0.154 | 0.301 |
|  | ln body size * tree | 0.003 | 0.172 | 0.016 | -0.310 | 0.332 | 0.987 |
|  |  |  |  |  |  |  |  |
| lumbar AEI ~ body size | |  |  |  |  |  |  |
|  |  | Estimate | StdErr | t.value | lowerbootCI | upperbootCI | p.value |
|  | intercept | 1.968 | 0.150 | 13.088 | 1.679 | 2.266 | 0.000 |
|  | ln body size | -0.090 | 0.038 | -2.395 | -0.162 | -0.016 | 0.019 |
|  |  |  |  |  |  |  |  |
| lumbar AEI ~ body size*ecotype | | |  |  |  |  |  |
|  |  | Estimate | StdErr | t.value | lowerbootCI | upperbootCI | p.value |
|  | intercept | 0.977 | 0.463 | 2.112 | 0.162 | 1.813 | 0.038 |
|  | ln body size | 0.214 | 0.149 | 1.441 | -0.053 | 0.468 | 0.153 |
|  | gliding | 0.383 | 0.532 | 0.720 | -0.603 | 1.333 | 0.473 |
|  | ground | 1.464 | 0.491 | 2.983 | 0.534 | 2.303 | 0.004 |
|  | tree | 0.755 | 0.502 | 1.505 | -0.185 | 1.687 | 0.136 |
|  | ln body size * gliding | -0.067 | 0.165 | -0.406 | -0.363 | 0.236 | 0.686 |
|  | ln body size * ground | -0.444 | 0.156 | -2.854 | -0.715 | -0.163 | 0.006 |
|  | ln body size * tree | -0.253 | 0.159 | -1.594 | -0.543 | 0.049 | 0.115 |
|  |  |  |  |  |  |  |  |
| sacral AEI ~ body size | |  |  |  |  |  |  |
|  |  | Estimate | StdErr | t.value | lowerbootCI | upperbootCI | p.value |
|  | intercept | 0.699 | 0.164 | 4.254 | 0.373 | 1.038 | 0.000 |
|  | ln body size | -0.011 | 0.044 | -0.252 | -0.104 | 0.080 | 0.802 |
|  |  |  |  |  |  |  |  |
| sacral AEI ~ body size*ecotype | | |  |  |  |  |  |
|  |  | Estimate | StdErr | t.value | lowerbootCI | upperbootCI | p.value |
|  | intercept | 0.448 | 0.797 | 0.562 | -1.029 | 1.968 | 0.576 |
|  | ln body size | 0.085 | 0.264 | 0.322 | -0.417 | 0.576 | 0.748 |
|  | gliding | -0.095 | 0.856 | -0.111 | -1.630 | 1.518 | 0.912 |
|  | ground | 0.368 | 0.824 | 0.446 | -1.169 | 1.852 | 0.657 |
|  | tree | 0.117 | 0.830 | 0.141 | -1.366 | 1.570 | 0.888 |
|  | ln body size * gliding | 0.030 | 0.277 | 0.110 | -0.495 | 0.533 | 0.913 |
|  | ln body size * ground | -0.099 | 0.270 | -0.366 | -0.587 | 0.392 | 0.716 |
|  | ln body size * tree | -0.073 | 0.272 | -0.267 | -0.562 | 0.420 | 0.790 |
|  |  |  |  |  |  |  |  |
| size-corrected rib length ~ body size | | |  |  |  |  |  |
|  |  | Estimate | StdErr | t.value | lowerbootCI | upperbootCI | p.value |
|  | intercept | 0.000 | 0.065 | 0.000 | -0.134 | 0.135 | 0.999 |
|  | ln body size | 0.000 | 0.017 | 0.000 | -0.034 | 0.035 | 0.999 |
|  |  |  |  |  |  |  |  |
| size-corrected rib length ~ body size*ecotype | | | |  |  |  |  |
|  |  | Estimate | StdErr | t.value | lowerbootCI | upperbootCI | p.value |
|  | intercept | 0.567 | 0.289 | 1.962 | 0.029 | 1.151 | 0.053 |
|  | ln body size | -0.173 | 0.096 | -1.809 | -0.367 | 0.005 | 0.074 |
|  | gliding | -0.438 | 0.310 | -1.410 | -1.063 | 0.179 | 0.163 |
|  | ground | -0.689 | 0.299 | -2.305 | -1.313 | -0.148 | 0.024 |
|  | tree | -0.590 | 0.301 | -1.960 | -1.216 | -0.006 | 0.054 |
|  | ln body size * gliding | 0.138 | 0.100 | 1.377 | -0.060 | 0.340 | 0.172 |
|  | ln body size * ground | 0.218 | 0.098 | 2.224 | 0.037 | 0.423 | 0.029 |
|  | ln body size * tree | 0.171 | 0.099 | 1.735 | -0.017 | 0.370 | 0.087 |

**Table S3**. Slope and intercept coefficients of PGLS models between body shape components, size, and ecotype. 95% bootstrap confidence intervals were used to determine if body size-shape relationships were allometric. Bolded values indicate slopes deviated from isometry. “All species” slopes and intercepts were obtained from PGLS regressions of body shape ~ body size whereas the ecotype specific slopes and intercepts were obtained from PGLS regressions with ANCOVA design of body shape ~ body size*ecotype.

|  | ecotype | intercept | intercept L95% | intercept U95% | slope | slope L95% | slope U95% |
| --- | --- | --- | --- | --- | --- | --- | --- |
| ln head-body elongation ratio | | |  |  |  |  |  |
|  | all squirrels | 1.73 | 1.56 | 1.90 | -0.02 | -0.07 | 0.02 |
|  | chipmunk | 0.9 | 0.27 | 1.58 | **0.24** | **0.01** | **0.45** |
|  | gliding | 1.27 | 1.03 | 1.50 | **0.12** | **0.06** | **0.19** |
|  | ground | 2.01 | 1.83 | 2.17 | **-0.11** | **-0.15** | **-0.06** |
|  | tree | 1.74 | 1.55 | 1.94 | -0.02 | -0.08 | 0.03 |
|  |  |  |  |  |  |  |  |
| ln head elongation ratio | | |  |  |  |  |  |
|  | all squirrels | 0.31 | 0.11 | 0.49 | **0.18** | **0.13** | **0.23** |
|  | chipmunk | 0.27 | -0.59 | 1.11 | 0.21 | -0.06 | 0.50 |
|  | gliding | 0.19 | -0.14 | 0.50 | **0.21** | **0.13** | **0.30** |
|  | ground | 0.17 | -0.07 | 0.38 | **0.22** | **0.16** | **0.29** |
|  | tree | 0.70 | 0.43 | 0.95 | 0.06 | -0.01 | 0.14 |
|  |  |  |  |  |  |  |  |
| ln cervical AEI | |  |  |  |  |  |  |
|  | all squirrels | 0.40 | 0.22 | 0.56 | **0.22** | **0.18** | **0.26** |
|  | chipmunk | -0.45 | -1.06 | 0.10 | **0.50** | **0.32** | **0.69** |
|  | gliding | 0.14 | -0.16 | 0.46 | **0.29** | **0.20** | **0.37** |
|  | ground | 0.69 | 0.49 | 0.91 | **0.13** | **0.07** | **0.18** |
|  | tree | 0.23 | -0.03 | 0.49 | **0.27** | **0.20** | **0.34** |
|  |  |  |  |  |  |  |  |
| ln thoracic AEI | |  |  |  |  |  |  |
|  | all squirrels | 1.94 | 1.65 | 2.21 | -0.06 | -0.13 | 0.02 |
|  | chipmunk | 1.72 | 0.85 | 2.68 | 0.01 | -0.30 | 0.29 |
|  | gliding | 1.46 | 0.97 | 1.97 | 0.12 | -0.01 | 0.26 |
|  | ground | 2.26 | 1.94 | 2.61 | **-0.17** | **-0.26** | **-0.09** |
|  | tree | 1.66 | 1.21 | 2.10 | 0.01 | -0.11 | 0.13 |
|  |  |  |  |  |  |  |  |
| ln lumbar AEI | |  |  |  |  |  |  |
|  | all squirrels | 1.97 | 1.68 | 2.27 | **-0.09** | **-0.16** | **-0.02** |
|  | chipmunk | 0.98 | 0.19 | 1.88 | 0.21 | -0.08 | 0.48 |
|  | gliding | 1.36 | 0.87 | 1.91 | **0.15** | **0.00** | **0.28** |
|  | ground | 2.44 | 2.11 | 2.78 | **-0.23** | **-0.31** | **-0.15** |
|  | tree | 1.73 | 1.30 | 2.12 | -0.04 | -0.15 | 0.08 |
|  |  |  |  |  |  |  |  |
| ln sacral AEI | |  |  |  |  |  |  |
|  | all squirrels | 0.7 | 0.38 | 1.01 | -0.01 | -0.09 | 0.08 |
|  | chipmunk | 0.45 | -0.98 | 1.88 | 0.09 | -0.39 | 0.56 |
|  | gliding | 0.35 | -0.24 | 0.95 | 0.12 | -0.04 | 0.28 |
|  | ground | 0.82 | 0.41 | 1.17 | -0.01 | -0.11 | 0.1 |
|  | tree | 0.57 | 0.16 | 0.99 | 0.01 | -0.11 | 0.13 |
|  |  |  |  |  |  |  |  |
| ln size-corrected rib length | | |  |  |  |  |  |
|  | all squirrels | 0.00 | -0.12 | 0.12 | 0.00 | -0.03 | 0.03 |
|  | chipmunk | 0.57 | 0.02 | 1.09 | -0.17 | -0.35 | 0.01 |
|  | gliding | 0.13 | -0.07 | 0.34 | -0.03 | -0.09 | 0.02 |
|  | ground | -0.12 | -0.27 | 0.02 | **0.04** | **0.01** | **0.09** |
|  | tree | -0.02 | -0.18 | 0.13 | 0.00 | -0.04 | 0.04 |

**Table S4.** Output tables of PGLS regressions between size-corrected limb lengths and body shape.

| ln size-corrected forelimb length ~ ln body shape (reduced) | | | | |  |  |  |
| --- | --- | --- | --- | --- | --- | --- | --- |
|  |  | Estimate | StdErr | t.value | lowerbootCI | upperbootCI | p.value |
|  | intercept | -0.134 | 0.223 | -0.602 | -0.573 | 0.337 | 0.549 |
|  | ln body shape | 0.082 | 0.132 | 0.617 | -0.188 | 0.340 | 0.539 |
|  |  |  |  |  |  |  |  |
| ln size-corrected forelimb length ~ ln body shape*ecotype (reduced) | | | | | |  |  |
|  |  | Estimate | StdErr | t.value | lowerbootCI | upperbootCI | p.value |
|  | intercept | -0.083 | 0.466 | -0.178 | -0.931 | 0.841 | 0.859 |
|  | ln body shape | -0.008 | 0.289 | -0.028 | -0.575 | 0.510 | 0.977 |
|  | gliding | -0.248 | 0.628 | -0.395 | -1.450 | 0.962 | 0.694 |
|  | ground | 0.573 | 0.526 | 1.090 | -0.417 | 1.537 | 0.280 |
|  | tree | 0.059 | 0.548 | 0.107 | -0.982 | 1.034 | 0.915 |
|  | ln body shape * gliding | 0.381 | 0.379 | 1.005 | -0.348 | 1.099 | 0.319 |
|  | ln body shape * ground | -0.382 | 0.326 | -1.172 | -0.980 | 0.228 | 0.245 |
|  | ln body shape * tree | 0.014 | 0.337 | 0.042 | -0.591 | 0.648 | 0.966 |
|  |  |  |  |  |  |  |  |
| ln size-corrected hindlimb length ~ ln body shape (reduced) | | | | | |  |  |
|  |  | Estimate | StdErr | t.value | lowerbootCI | upperbootCI | p.value |
|  | intercept | -0.128 | 0.182 | -0.703 | -0.506 | 0.259 | 0.484 |
|  | ln body shape | 0.078 | 0.107 | 0.725 | -0.145 | 0.291 | 0.471 |
|  |  |  |  |  |  |  |  |
| ln size-corrected hindlimb length ~ ln body shape*ecotype (reduced) | | | | | |  |  |
|  |  | Estimate | StdErr | t.value | lowerbootCI | upperbootCI | p.value |
|  | intercept | 0.005 | 0.349 | 0.014 | -0.685 | 0.668 | 0.989 |
|  | ln body shape | -0.050 | 0.211 | -0.237 | -0.441 | 0.360 | 0.814 |
|  | gliding | -0.354 | 0.673 | -0.526 | -1.558 | 0.923 | 0.601 |
|  | ground | -0.140 | 0.436 | -0.321 | -0.935 | 0.667 | 0.749 |
|  | tree | 0.203 | 0.495 | 0.410 | -0.724 | 1.167 | 0.683 |
|  | ln body shape * gliding | 0.377 | 0.393 | 0.960 | -0.351 | 1.085 | 0.341 |
|  | ln body shape * ground | 0.073 | 0.267 | 0.272 | -0.419 | 0.562 | 0.786 |
|  | ln body shape * tree | -0.088 | 0.296 | -0.296 | -0.661 | 0.457 | 0.768 |
|  |  |  |  |  |  |  |  |
| ln size-corrected forelimb length ~ ln body shape (full) | | | | |  |  |  |
|  |  | Estimate | StdErr | t.value | lowerbootCI | upperbootCI | p.value |
|  | intercept | 0.053 | 0.183 | 0.290 | -0.310 | 0.415 | 0.773 |
|  | ln body shape | -0.032 | 0.110 | -0.295 | -0.252 | 0.189 | 0.769 |
|  |  |  |  |  |  |  |  |
| ln size-corrected forelimb length ~ ln body shape*ecotype (full) | | | | | |  |  |
|  |  | Estimate | StdErr | t.value | lowerbootCI | upperbootCI | p.value |
|  | intercept | -0.040 | 0.367 | -0.109 | -0.689 | 0.656 | 0.913 |
|  | ln body shape | -0.016 | 0.227 | -0.069 | -0.448 | 0.389 | 0.945 |
|  | gliding | -0.025 | 0.555 | -0.045 | -1.038 | 0.969 | 0.965 |
|  | ground | 0.544 | 0.427 | 1.274 | -0.220 | 1.328 | 0.208 |
|  | tree | 0.146 | 0.474 | 0.308 | -0.752 | 1.037 | 0.760 |
|  | ln body shape * gliding | 0.165 | 0.335 | 0.491 | -0.441 | 0.776 | 0.625 |
|  | ln body shape * ground | -0.348 | 0.264 | -1.316 | -0.831 | 0.128 | 0.194 |
|  | ln body shape * tree | -0.039 | 0.290 | -0.136 | -0.588 | 0.517 | 0.892 |
|  |  |  |  |  |  |  |  |
| ln size-corrected hindlimb length ~ ln body shape (full) | | | | |  |  |  |
|  |  | Estimate | StdErr | t.value | lowerbootCI | upperbootCI | p.value |
|  | intercept | -0.065 | 0.194 | -0.333 | -0.419 | 0.302 | 0.741 |
|  | ln body shape | 0.040 | 0.116 | 0.341 | -0.183 | 0.252 | 0.735 |
|  |  |  |  |  |  |  |  |
| ln size-corrected hindlimb length ~ ln body shape*ecotype (full) | | | | | |  |  |
|  |  | Estimate | StdErr | t.value | lowerbootCI | upperbootCI | p.value |
|  | intercept | 0.143 | 0.378 | 0.379 | -0.569 | 0.891 | 0.707 |
|  | ln body shape | -0.114 | 0.231 | -0.491 | -0.564 | 0.320 | 0.626 |
|  | gliding | -1.416 | 0.804 | -1.761 | -2.937 | 0.090 | 0.084 |
|  | ground | -0.065 | 0.469 | -0.138 | -1.011 | 0.829 | 0.891 |
|  | tree | -0.164 | 0.542 | -0.302 | -1.225 | 0.842 | 0.764 |
|  | ln body shape * gliding | 0.966 | 0.479 | 2.020 | 0.069 | 1.887 | 0.049 |
|  | ln body shape * ground | 0.005 | 0.289 | 0.016 | -0.560 | 0.582 | 0.988 |
|  | ln body shape * tree | 0.126 | 0.331 | 0.382 | -0.492 | 0.760 | 0.704 |

**Table S5**. Slope and intercept coefficients of PGLS models between limb length and body shape. 95% bootstrap confidence intervals were used to determine if body size-shape relationships were allometric. Bolded values indicate slopes deviated from isometry. “All species” slopes and intercepts were obtained from PGLS regressions of size-corrected limb length ~ body shape whereas the ecotype specific slopes and intercepts were obtained from PGLS regressions with ANCOVA design of size-corrected limb length ~ body shape*ecotype.

|  | ecotype | intercept | intercept L95% | intercept U95% | slope | slope L95% | slope U95% |
| --- | --- | --- | --- | --- | --- | --- | --- |
| forelimb_long vs body shape | | |  |  |  |  |  |
|  | all squirrels | -0.13 | -0.56 | 0.28 | 0.08 | -0.17 | 0.34 |
|  | chipmunk | -0.08 | -0.99 | 0.82 | -0.01 | -0.57 | 0.56 |
|  | gliding | -0.33 | -1.11 | 0.42 | 0.37 | -0.07 | 0.82 |
|  | ground | 0.49 | 0.07 | 0.92 | **-0.39** | **-0.66** | **-0.13** |
|  | tree | -0.02 | -0.50 | 0.52 | 0.01 | -0.33 | 0.29 |
|  |  |  |  |  |  |  |  |
| hind limb_long vs body shape | | |  |  |  |  |  |
|  | all squirrels | -0.13 | -0.50 | 0.24 | 0.08 | -0.14 | 0.29 |
|  | chipmunk | 0.01 | -0.63 | 0.69 | -0.05 | -0.46 | 0.34 |
|  | gliding | -0.35 | -1.41 | 0.74 | 0.33 | -0.31 | 0.94 |
|  | ground | -0.14 | -0.65 | 0.39 | 0.02 | -0.3 | 0.34 |
|  | tree | 0.21 | -0.43 | 0.86 | -0.14 | -0.54 | 0.25 |
|  |  |  |  |  |  |  |  |
| forelimb_full vs body shape | | |  |  |  |  |  |
|  | all squirrels | 0.05 | -0.29 | 0.44 | -0.03 | -0.27 | 0.17 |
|  | chipmunk | -0.04 | -0.74 | 0.68 | -0.02 | -0.47 | 0.42 |
|  | gliding | -0.06 | -0.8 | 0.72 | 0.15 | -0.31 | 0.59 |
|  | ground | 0.5 | 0.12 | 0.91 | **-0.36** | **-0.62** | **-0.13** |
|  | tree | 0.11 | -0.47 | 0.66 | -0.06 | -0.39 | 0.29 |
|  |  |  |  |  |  |  |  |
| hind limb_full vs body shape | | |  |  |  |  |  |
|  | all squirrels | -0.06 | -0.46 | 0.33 | 0.04 | -0.21 | 0.27 |
|  | chipmunk | 0.14 | -0.55 | 0.92 | -0.11 | -0.59 | 0.29 |
|  | gliding | -1.27 | -2.71 | 0.07 | **0.85** | **0.04** | **1.71** |
|  | ground | 0.08 | -0.46 | 0.65 | **-0.11** | **-0.46** | **0.22** |
|  | tree | -0.02 | -0.78 | 0.73 | 0.01 | -0.43 | 0.49 |
